# Atmospheric blocking and intercomparison of objective detection methods: flow field characteristics

**DOI:** 10.1007/s00382-019-04782-5

**Published:** 2019-04-25

**Authors:** M. C. Pinheiro, P. A. Ullrich, R. Grotjahn

**Affiliations:** 1 Department of Land, Air, and Water Resources, University of California, Davis, 1 Shields Ave, Davis, CA USA; 2 Lawrence Berkeley National Laboratory, 1 Cyclotron Rd, Berkeley, CA USA

**Keywords:** Blocking, Objective detection, Climate variability, Climatology

## Abstract

Objective methods for identifying and quantifying atmospheric blocking have been developed over recent decades, primarily targeting North Atlantic blocks. Differences arise from these methods, leading to changes in the resultant blocking climatology. To understand these differences, and better inform future assessments built on quantitative detection of blocks, this paper examines blocking properties produced by three different objective detection algorithms over the global extratropics. Blocking criteria examined include 500 hPa geopotential height anomaly ($Z^*$), column-averaged potential vorticity anomaly ($PV^*$), and 500 hPa geopotential height gradient (*AGP*). Results are analyzed for blocking climatologies and for instantaneous blocking patterns, as well as distributions of block size, speed, duration, and distance traveled. The results emphasize physical characteristics of the flow field and the subsequent blocking regions that emerge; overall, $PV^*$ and $Z^*$ blocked regions often have higher pattern correlation and spatial similarity, though these two methods also display high agreement with *AGP* in some instances.$Z^*$ finds the largest (and greatest number of) blocked regions, while $PV^*$-detected regions are smallest in all instances except Southern Hemisphere winter. In some cases, $PV^*$ tracks a nearby jet streak, leading to differences with height-based algorithms. All three algorithms detect some questionable low-latitude blocks that are stationary and persist but do not impair zonal flow, although at different times. Therefore, careful consideration of the algorithm biases is important in future blocking studies. For example, linking extreme weather to detected blocking could vary substantially depending on the algorithm used.

## Introduction

Atmospheric blocking is a synoptic-scale weather phenomenon with important social and ecological impacts that arise due to its correlation with many kinds of extreme weather, such as heat waves (Pfahl and Wernli 2012; Grotjahn 2011; Lee and Grotjahn 2015), cold spells (Sillmann et al. 2011; Grotjahn and Faure 2008), and floods (Houze et al. 2011; Hong et al. 2011). Yet it is a phenomenon that is not fully understood in terms of the underlying physics. The American Meteorological Society (AMS) definition of blocking (Glickman 2000) uses three criteria for classifying a flow pattern as blocked:

1. persistent obstruction of the normal west-to-east flow pattern,
2. pronounced meridional flow in the upper levels, and
3. anticyclonic circulation at high latitudes accompanying cyclonic circulation at low latitudes.

The onset of a blocking feature results in temporary redirection of the jet stream, which is in turn responsible for the aforementioned anomalous weather conditions. Blocks take several forms, including the well-known omega block, a poleward high co-located between two equatorward lows; high/low dipoles; and persistent ridges, and often take on more than one form during their life cycles. All of these varieties nonetheless satisfy the criteria established by the AMS definition.

Blocking has been studied for decades, but the first attempts at finding blocks required visual inspection of flow patterns and were thus limited in scope (Rex 1950). Such subjective assessment leads different individuals to potentially different conclusions about a flow pattern. The development of an objective procedure for identifying blocks was thus important for a number of reasons:

1. *Consistency* While the development of objective methods requires some preliminary human judgment in the choice of parameters, an algorithmic definition of blocking removes the human subjectivity from the rest of the procedure and thus produces results that are internally consistent for a given dataset.
2. *Efficiency* These methods can be automated, making computation and comparison of blocking climatologies possible across very large volumes of high-resolution, multi-decadal data.
3. *Improved scientific understanding* Algorithms are based on current concepts of block formation and maintenance. Objective detection methods can allow for these concepts to be rigorously tested and improved as more information is gathered.

Objective methods based on a variety of fields and techniques have been developed over the years (see Figure 1 of Barriopedro et al. 2010 for an overview), and intercomparison studies such as Barnes et al. (2012) have explored whether these methods produce consistent blocking climatologies. Barnes et al. (2012) compares three longitudinally-varying (1D) methods (i.e. calculated about a single time-varying central latitude): Pelly and Hoskins 2003 (potential temperature ($\theta$) on a constant potential vorticity surface), Tibaldi and Molteni 1990 (500 hPa geopotential height (*Z*500) gradient over a latitudinal range), and Scaife et al. 2010 (zonal wind over a latitudinal range). The analysis, which was performed on 43 years of Northern Hemisphere data, concludes that these methods yield similar results in terms of calculated blocking frequency and duration across the time and longitude axes. However, these are only two possible metrics under which objective methods can be examined, and other papers have noted inherent differences in the methods due to both the data and the chosen method. For example, Davini et al. (2012) notes that there are distinct regional differences in both the geopotential height fields and the resultant characteristics of detected blocks. Over Greenland, blocks principally correspond to cyclonic Rossby wave breaking with a dipole structure, and split-flow blocking generally happen in the midlatitudes over central Europe. The structure of a block impacts the effectiveness of the detection method; Scherrer et al. (2006) compared detection of an omega block versus a persistent ridge, using the aforementioned *Z*500 gradient method of Tibaldi and Molteni (1990) as well as two potential vorticity (*PV*)-based metrics. All three detection methods produced similar results for the omega block, but displayed notable difference in both the size and center locations of the blocked areas for the ridge. The authors attribute the differences to both the choice of the variable (*Z*500 versus *PV*) and the use of an anomaly versus total field.

This study expands upon previous intercomparison efforts; blocking is assessed in terms of distinct blocking features rather than per gridpoint, and each algorithm is applied across the full latitude-longitude (2D) range of the study regions, which include both the Northern (NH) and Southern (SH) Hemisphere midlatitudes. Assessing algorithmic differences via individual blocking events allows determination of block characteristics beyond blocking frequency: here, we consider the size, duration, distance traveled, and zonal speed of each block as determined by each algorithm. We choose to utilize 2D rather than 1D blocking indices in order to more fully examine regional variations in blocking; most notably, low-latitude blocking is often missed by 1D methods. Furthermore, it demonstrates how these algorithms perform in regional climatologies outside of those for which they were developed. In particular, attention is paid to the underlying flow patterns that lead to differences in objective blocking climatologies. Our analysis shows that each of the assessed algorithms only capture a subset of meteorological patterns defined by the AMS definition of blocking, and the level of agreement between algorithms is highly dependent on region and block type. This an important point to consider when attempting to assess current and future blocking trends and the impacts of corresponding extreme weather. A further benefit of this study is that the metrics and algorithms developed through this work may be leveraged for evaluation of global climate datasets, either from individual model runs or from coordinate intercomparison efforts (for details, see “Appendix B”).

Section 2 outlines the three objective detection algorithms and the analysis framework, which was developed with the goal of standardizing the detection methodology as much as possible across the algorithms. Section 3 compares results between the three algorithms in terms of both the averaged and instantaneous blocking patterns, as well as some of the characteristics of the detected blocks. Section 4 explains some of the meteorological factors which influence the algorithms’ results, and Section 5 summarizes and discusses the implications of differences between algorithm results.

## Data and methodology

### Data

Our dataset is the ERA-Interim reanalysis from the European Center for Medium-Range Weather Forecasts (Dee et al. 2011). Temperature, meridional and zonal wind, and geopotential variables are 6-h at 1$^\circ$ spatial resolution in the time period of March 1, 1979–February 28, 2018 (39 years). The latitude range employed spans 25$^\circ$–75$^\circ$ in each hemisphere, and the longitudinal extents of each region are outlined in Table 1; the abbreviations in the table will be used for each region hereafter. These regions are based on the suggested ranges in Wiedenmann et al. (2002); each region is roughly centered over a local maximum of blocking frequency.

**Table 1:** Longitudinal extents of study regions; each region has a latitudinal extent of 25$^\circ$–75$^\circ$ in their respective hemispheres

NH	Continent (NC)	Pacific (NP)	Atlantic (NA)
	40E, 140E	140E, 100W	100W, 40E
SH	Indian Ocean (SI)	Pacific (SP)	Atlantic (SA)
	30E, 130E	130E, 60W	60W, 30E

The regions can be seen outlined on the maps in Figs. 2, 3, 4, 5 and 6. The two-letter abbreviations will be used to refer to these regions throughout the paper

Two of the methods (Tibaldi and Molteni (1990), hereafter referred to as TM90; and Dole and Gordon (1983), hereafter referred to as DG83) are based on the *Z*500 variable, while the third method (Schwierz et al. 2004, hereafter referred to as S04) is based on vertically averaged PV (*VPV*). The *Z*500 fields are derived from the geopotential variable ($Z = \varPhi / g$). From temperature and the horizontal wind components, we calculated Ertel PV (*EPV*, described in “Appendix A”) and then averaged this over the 150–500 hPa layer to produce *VPV*.

### Blocking detection methods

The blocking climatology varies with the choice of detection scheme, as shown below. In order to explore some of the points raised by Davini et al. (2012) and Scherrer et al. (2006)—particularly the differences due to variable choice and region—we utilize schemes that are based on two different variables (*Z*500 and *VPV*) and field types (anomaly-based versus total field).

**Fig. 1:**
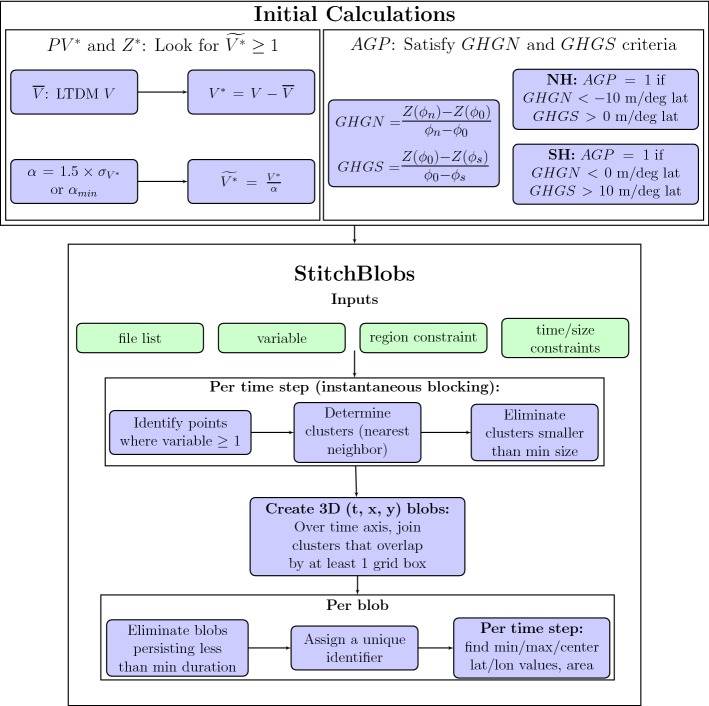
Schematic of workflow for blocking calculations using StitchBlobs

A standardized analysis framework, StitchBlobs, was developed for the intercomparison of global blocking detection schemes. The blocking detection workflow is outlined in Fig. 1, and further details on StitchBlobs, which is part of the TempestExtremes package (Ullrich and Zarzycki 2017), are provided in “Appendix B”. Instantaneous blocks that meet the spatial constraint (minimum area of $10^6$ km$^2$) are stitched together across time into distinct blocking events. StitchBlobs identifies events that fit the minimum time constraint (5 days, the characteristic length of persistent height anomalies as per DG83), then tags each event with a unique identifier and provides per-time-step information on each block’s location (in terms of block center latitude/longitude coordinates) and size (in terms of either maximum latitude/longitude extent or the area of the cluster). This allows users to follow individual blocking events from formation to dissipation, and examine seasonal and regional trends in block characteristics such as size and zonal distance traveled on a per-block basis.

The methods are briefly summarized in Table 2, and explained in greater detail in the following sections. In order to differentiate between the methods from the original papers and the ones presented here, we use different abbreviations: our version of TM90 is *AGP*, DG83 is $Z^*$, and S04 is $PV^*$.

**Table 2:** Summary of original methods and modifications

Method	Tibaldi and Molteni (1990) (*AGP*)	Dole and Gordon (1983) ($Z^*$)	Schwierz et al. (2004) ($PV^*$)
Variable	*Z*500 latitudinal gradient (*GHGN*, *GHGS*)	Z500 anomaly ($Z500^*$) with latitudinal scaling factor	Vertically averaged PV anomaly ($VPV^*$)
Detected feature	Change in *Z*500 $15^\circ$ above/below point, implying presence of high	Positive $Z500^*$ with respect to climatological mean	Reversal of flow with respect to climatological mean
Original blocking criteria	$\hbox {GHGN}<-10\,\hbox {m/deg}$ lat and $\hbox {GHGS}>0\,\hbox {m/deg}$ lat over 4 days, per gridpoint	$Z500^*\ge 100$ m over 10 days, per gridpoint	$VPV^*\le -1.2$ PVU, with at least 70% overlap between contours over 5 days
Change to original method	Extend analysis to all latitudes within $25^{\circ }$–$75^{\circ }$, 5 days’ persistence with contour overlap	Varying anomaly threshold, 5 days’ persistence with contour overlap	Varying anomaly threshold with positive sign for SH

#### Geopotential height gradient (AGP)

The most frequently cited blocking detection method in the literature is TM90, which is itself based on Lejenäs and Økland (1983). Two gradients are calculated about a central latitude as follows:

1 $$\begin{aligned} GHGN= & \frac{Z500(\phi _n)-Z500(\phi _0)}{\phi _n-\phi _0} \end{aligned}$$

2 $$\begin{aligned} GHGS= & \frac{Z500(\phi _0)-Z500(\phi _s)}{\phi _0-\phi _s} \end{aligned}$$

where $\phi _0$, $\phi _n$, and $\phi _s$ are the reference latitude and the latitudes 20$^\circ$ above and below $\phi _0$, respectively, and *GHGN* and *GHGS* are the height gradients. For $GHGN<-10$ m/deg lat and $GHGS>0$ m/deg lat, the point is considered instantaneously blocked; the negative *GHGN* and positive *GHGS* values imply a large-scale high in the 500 hPa geopotential height field.

TM90 performed these calculations about a single reference latitude band ($60^\circ \hbox {N}$), and Barnes et al. (2012) performed these calculations about a varying central latitude. The TM90 algorithm was modified by Scherrer et al. (2006) to extend analysis to latitudes 35$^\circ \hbox {N}$–75$^\circ \hbox {N}$ and define $\phi _n$ and $\phi _s$ as $15^\circ$ away from $\phi _0$; we further extend the analysis to $25^\circ$ in both hemispheres. Furthermore, to apply this method in the SH, it is necessary to switch the criteria and signs for the two gradients, since the orientation of ridges is flipped and the SH latitudes are negative. Therefore, in *AGP*, $GHGN<0$ m/deg lat and $GHGS>10$ m/deg lat in the SH.

In TM90, a blocking episode is defined as a region of blocked flow that extends over at least 12$^\circ$ longitude for a minimum of 4 days. This satisfies the second and third points of the AMS definition (meridional flow and anticyclonic circulation); however, the fact that this method is based on total fields means that it does not necessarily satisfy the first point (obstruction of normal flow), since there is no reference to the mean climatology.

#### Geopotential height anomaly ($\hbox {Z}^*$)

DG83 utilizes *Z*500 anomaly ($Z500^*$), which is first calculated as the height departure from the long term seasonal average, $h'$, then normalized by a latitudinal coefficient:

3 $$\begin{aligned} Z500^*= \left( \frac{\sin 45^\circ }{\sin \phi }\right) h'. \end{aligned}$$

This modification is necessitated by the latitudinal change in planetary vorticity; the conservation of absolute vorticity means that there must be an increase in relative vorticity due to the decrease in planetary vorticity in the poleward direction. At the higher latitudes, the convergence of latitudes leads to a bias in the representation of meridional energy propagation.

A single grid point is defined as blocked if $Z500^*$ exceeds 100 m for 10 days, although subsequent papers have used different combinations of heights and durations (for a 5-day minimum duration, Sausen et al. 1995 used 250 m). As with TM90, this detection method works by searching for high geopotential heights, although in this case the high is defined with respect to the long term average. DG83 is theoretically capable of satisfying the AMS criteria for blocking because anomalously high *Z*500 will modify the flow pattern in a manner consistent with all three requirements. With that said, the relationship between the climatological mean and the instantaneous field can lead to overprediction of blocking (particularly in the SH), as discussed in Sects. 4.1 and 4.3.

#### Potential vorticity anomaly (PV$^*$)

**Fig. 2:**
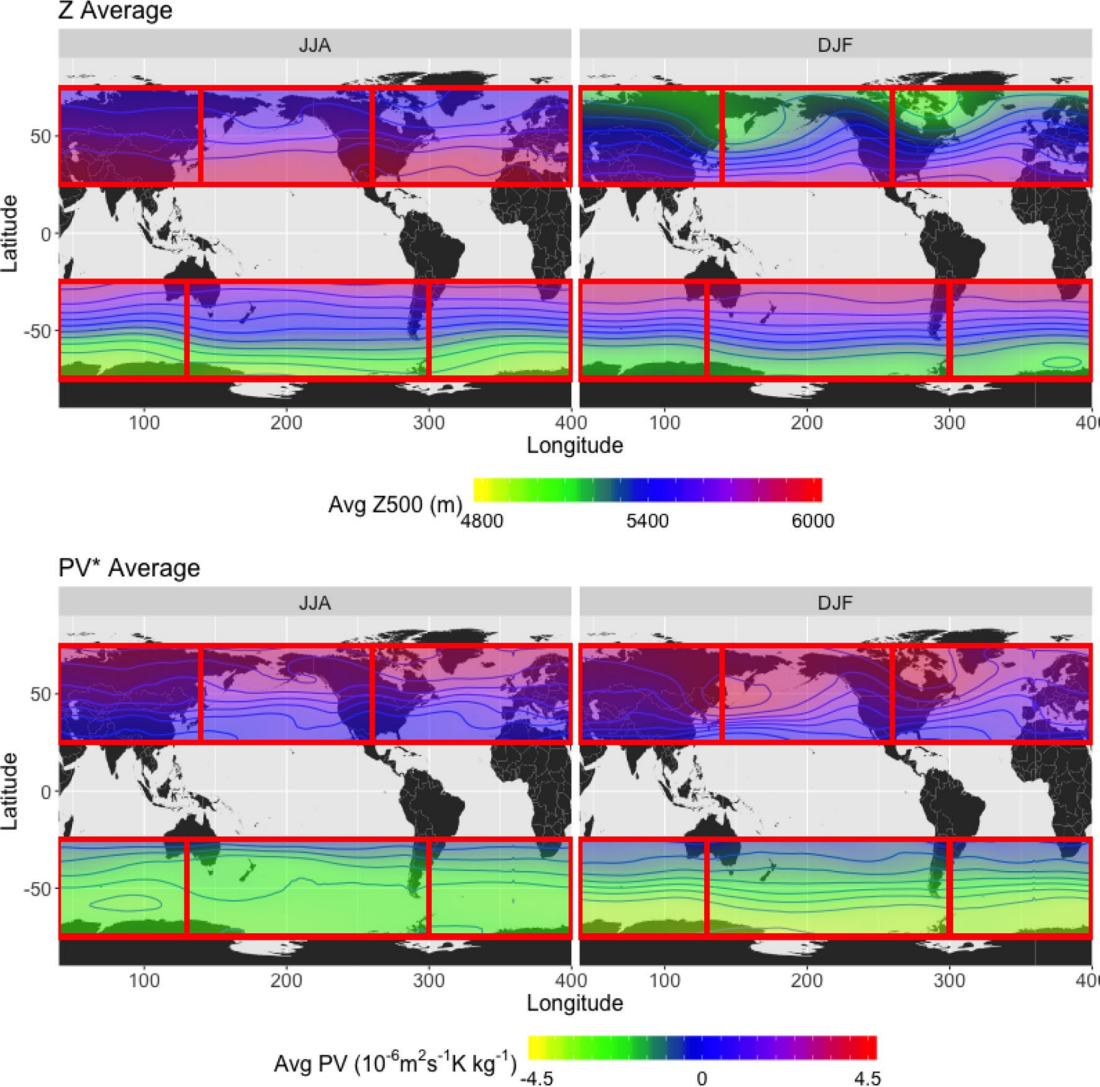
Seasonal averages of long term daily mean Z500 (top) and vertically averaged PV (bottom) values for (left) JJA, and (right) DJF, ERA-Interim 1979–2018. Each seasonal average contains 39 years’ worth of data. Red rectangles denote the study regions as outlined in Table 1; from left to right, the NH regions are NC, NP, and NA, and the SH regions are SI, SP, and SA. The contours are in intervals of 100 m for Z500 and 0.5 PVU for PV

S04 proposes a blocking detection method which entails searching for regions of persistent column-averaged (150–500 hPa) negative PV anomalies ($VPV^*$) in the NH (in $PV^*$, the relevant anomalies are positive in the SH). As with DG83, anomalies are calculated as instantaneous departures from a long term daily mean (defined as the 15-year monthly mean in S04). S04 favors the use of *VPV* over *Z*500 for anomaly-based detection, as $VPV^*$ more closely follows the shape of the dynamic tropopause, compared to a similarly situated *Z*500 anomaly (Figures 1b and 2b in S04). While S04 does not explicitly account for easterly flow as in TM90, the negative sign indicates anticyclonic circulation at and below the layer with easterlies countering the mean flow, thus signifying underlying higher pressure as in point 3 of the AMS definition. The use of $VPV^*$ also accounts somewhat for parts 1 and 2 of the AMS definition, but strongly negative (positive) values of vorticity in the NH (SH), discussed in Sect. 4.2, or relatively low values of *VPV* with respect to the climatological mean, discussed in Sect. 4.3, can cause this method to mistakenly identify unobstructed flow as blocked.

#### Modifications to anomaly methods

For the purposes of global intercomparison, a few minor modifications are needed for the anomaly-based detection methods. Since DG83 and S04 use different definitions of climatological means and thresholds, it is necessary to redefine these quantities using a consistent methodology rather than those of the original algorithms. The choice of threshold is an important consideration when applying a method to a new dataset; as DG83 and S04 were initially developed using data from NA DJF, their hardcoded thresholds are not directly applicable in other sectors. The applicability of threshold values to new datasets is also an important consideration for multi-model intercomparisons; Woollings et al. (2018) found that the timing and spatial distribution of projected changes in blocking using CMIP5 model output were dependent on region and methodology. Therefore, a constant threshold that was determined using one model may not be representative of the climatology of another model. For a unified global study such as this one, a constant threshold definition will lead to either under- or over-detection of blocks in other regions because the anomaly thresholds are calibrated to the climatology of that region and time period. Here we follow Barriopedro et al. (2010) and Dunn-Sigouin et al. (2012), who address this problem by replacing the constant threshold definition with one derived from the standard deviation of anomaly values.

*Long term daily mean and anomalies* For this study, mean fields are computed for each of the 365 days in a year (excluding leap days) across 39 years. The values on these 365 days are Fourier transformed and only the first 6 harmonics (0–5, with 0 corresponding to the mean and 5 corresponding to a 73 day span, or slightly less than a season) are used to back transform and obtain the long term daily mean (LTDM, denoted here as $\overline{V}$, the average of instantaneous variable *V*). This procedure is further explained in Grotjahn and Zhang (2017); it serves to smooth out the mean, which can otherwise have excessive day-to-day variations. Figure 2 shows seasonal averages of the LTDM for the *Z*500 and *VPV* fields. Anomalies from the LTDM, $V^*$, can thus be defined as

4 $$\begin{aligned} V^* = V-\overline{V}. \end{aligned}$$

*Threshold and normalized anomalies* Whereas DG83 and S04 use constant minimum anomaly value threshold values in their blocking definitions (thresholds of magnitude 100 m and 1.2 PVU, respectively), we calculate a threshold field that varies in both space and time. At each grid point in the relevant field, we calculate the standard deviation of that grid point’s time series; the threshold is defined as 1.5 times the standard deviation. These values are then smoothed in both the zonal and meridional directions in a manner similar to the LTDM computation. This procedure is then repeated across time with the first 6 harmonics as with the LTDM calculation. However, an additional minimum threshold criterion, $\alpha _{min}$ (100 m for $Z^*$ and 1.1 PVU for $PV^*$), is imposed. This is necessary for regions with little to no blocking activity, where the anomaly values (and thus the standard deviation and threshold values) are very low. Using the standard deviation of the anomalies for each day over the course of the 26-year time period, $\sigma _{V^*}$, threshold $\alpha$ is defined as

5 $$\begin{aligned} \alpha = {\left\{ \begin{array}{ll} 1.5\times \sigma _{V^*},&\quad \text {if } \alpha \ge \alpha _{min}\\ \alpha _{min}, &\quad \text {otherwise}. \end{array}\right. } \end{aligned}$$

**Fig. 3:**
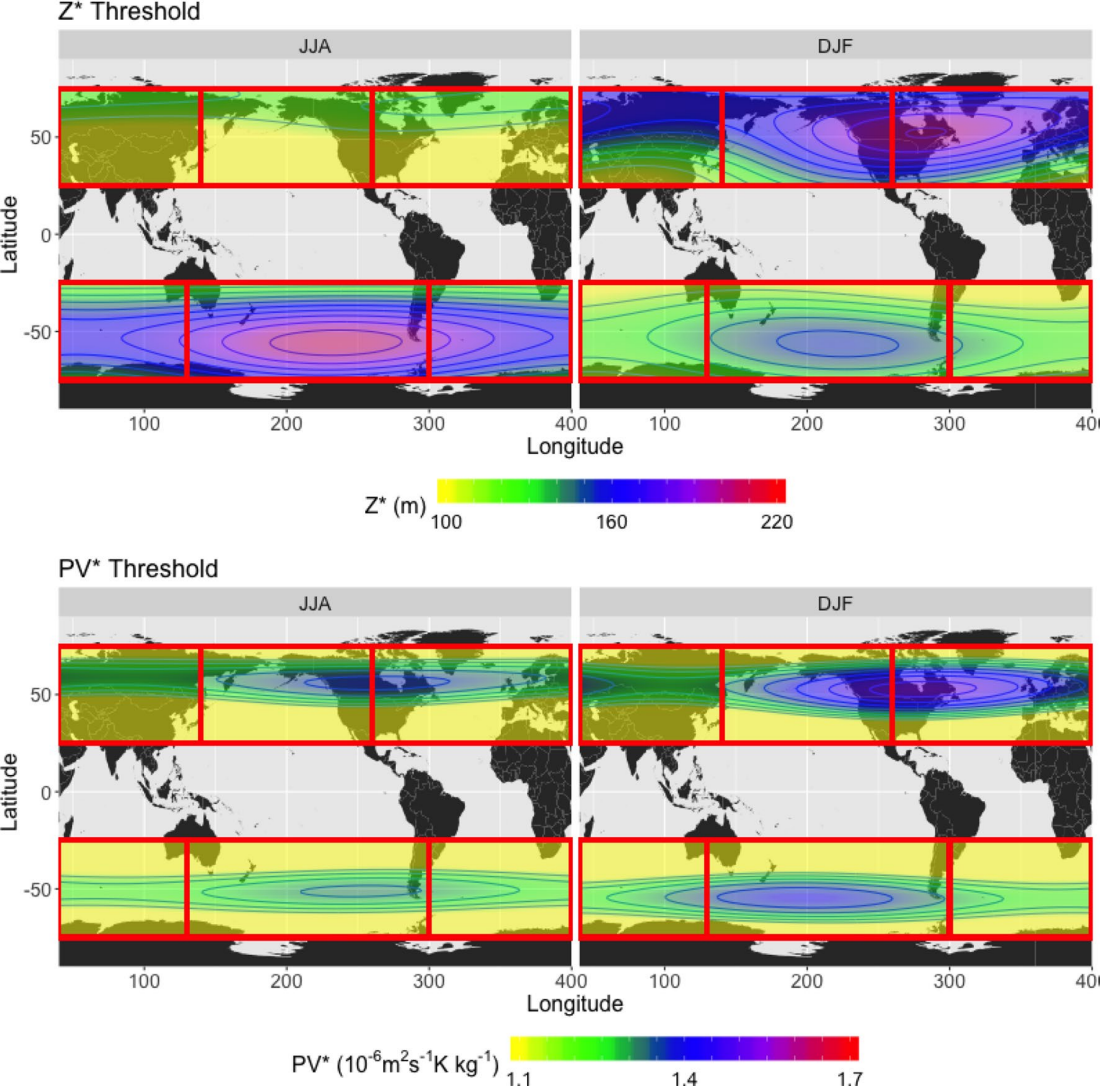
Seasonal averages of (top) Z500 anomaly ($Z500^*$) and (bottom) vertically averaged PV anomaly ($VPV^*$) threshold values, as described in Sect. 2.2.4, for (left) JJA and (right) DJF. Red rectangles denote same regions described in Fig. 2 caption. The contours are in intervals of 10 m for $Z500^*$ and 0.05 PVU for $VPV^*$

The range of threshold values as defined by the standard deviation is highly dependent on region and season, as apparent in Fig. 3. DG83 noted that the distribution of persistent $Z500^*$ values varied from region to region in the wintertime, with standard deviations of 170–180 m for the Atlantic and Pacific regions, as opposed to 45 m for Eastern Asia; it is evident that using a constant threshold on a global analysis is not advisable for $Z^*$. The distribution of $VPV^*$ values, while less drastically variable with respect to the magnitude of values compared to $Z500^*$, also displays some regional and seasonal differences with respect to anomaly magnitudes, particularly when comparing summer to winter. The spatiotemporally varying, standard deviation-based definition ensures that only anomaly values that are at the tails of the local distribution at that particular time step will be classified as blocked, irrespective of region or season.

The normalized anomalies, $\widetilde{V^*}$, are calculated as

6 $$\begin{aligned} \widetilde{V^*} = \frac{V^*}{\alpha }, \end{aligned}$$

and areas with $\widetilde{V^*}\ge 1$ are considered blocked.

### Quantification of agreement between methods

Three metrics are used for quantifying agreement between methods: pattern correlation between the seasonally-averaged blocking patterns, probability of co-occurrence, and block spatial similarity. We utilized multiple methods in order to separate out seasonally averaged versus instantaneous agreement; pattern correlation is useful for assessing the blocking climatology, but similarity and probability metrics provide further insight into the agreement between methods with regards to individual blocks. The quantification of probability and similarity serve similar purposes, but inevitably are different metrics for comparing detection algorithms. Two methods could have a high probability of co-occurrence if they consistently detect the same features, but a lesser value of similarity if the resultant clusters produced by the two methods are very different in size and shape.

*Pearson pattern correlation* This metric, denoted by *C*(*M*1, *M*2), measures the strength of linear relationships between frequency values at corresponding coordinates for methods *M*1 and *M*2. Higher correlation is seen when patterns are more similar, regardless of the relative magnitudes of the two data points. Pattern correlation has a range of possible values from − 1 to 1, in which magnitudes of 0.3 and below are considered weak, and strong at 0.7 and above. Negative values indicate an inverse relationship between *M*1 and *M*2. Centered pattern correlation is computed using the NCL pattern_cor function with cosine latitude weighting.

*Probability of co-occurrence* This metric, denoted by *P*(*M*1|*M*2), quantifies the likelihood that a block will be detected by method *M*1, given that method *M*2 also detects it. The methodology for calculating probability of co-occurrence can be found in “Appendix C”.

*Spatial similarity* This metric, denoted by *S*(*M*1, *M*2), quantifies the match between areas designated as part of a block by methods *M*1 and *M*2. *S* is the intersection divided by the union of the two areas. Since *S* varies for each block, an interquartile range will be presented in the results. The methodology for calculating spatial similarity can be found in “Appendix D”.

## Evaluation and intercomparison of atmospheric block metrics

We begin with a single case study, to highlight the importance of selecting a method, then present results for the entire ERA-Interim period in subsequent sections. The full ERA-Interim results for JJA and DJF are presented as follows. First, each algorithm is assessed in terms of its individual statistics (averaged blocking climatology, block duration, size, distance traveled, and zonal speed measurements). Agreement between detection methods (in terms of both averaged and instantaneous detection of blocks) is also assessed. Results are considered statistically significant if there is a meaningful relationship in the data that could not have occurred from random chance or sampling error alone. The methodology for establishing statistical significance for all of the metrics in Sect. 3 is explained in “Appendix E”. These metrics demonstrate that agreement in blocking frequency does not imply consistency in the character of individual blocks. Namely, one needs to be careful when drawing conclusions based on the blocking climatology alone, since there may be significant differences in the meteorological character of individual blocks. Further, these results are indicative that conclusions drawn with one blocking detection scheme may not hold when another algorithm is used.

### Case study: the ridiculously resilient ridge

We present a case study of a persistent and pronounced ridge pattern that repeatedly appeared off the western coast of North America in late 2013, then reoccurred during the winters of 2014–2015 and 2015–2016. This feature, dubbed the “Ridiculously Resilient Ridge” (or RRR for short) by Swain et al. (2014), was responsible for redirecting moisture-heavy air northwards during the winter months. Because California receives the bulk of its precipitation from December to March, the RRR was a key player in the drought that devastated the state for almost 6 years.

**Fig. 4:**
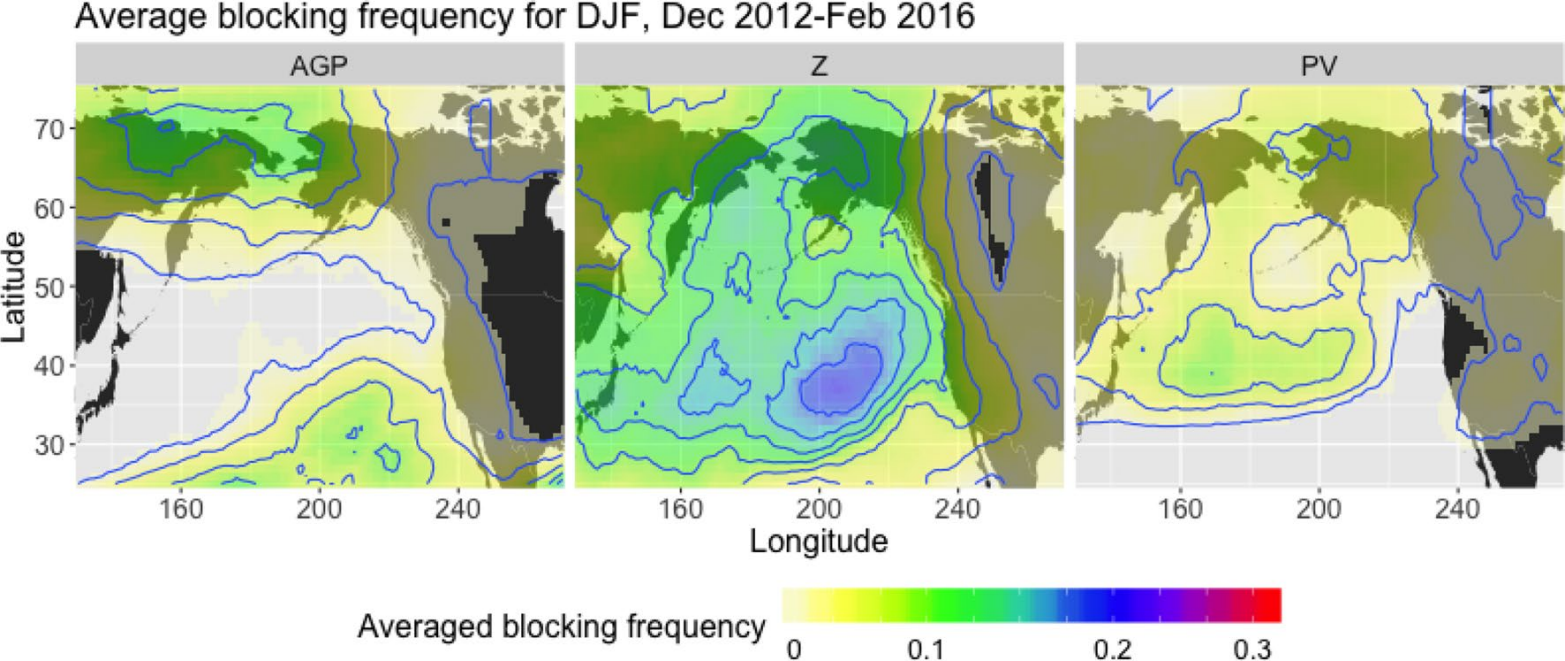
Blocking frequency, averaged over winters from 2012–2016, for the *AGP* method (left), $Z^*$ method (center), and $PV^*$ method (right)

**Table 3:** Pattern correlation, co-occurrence, and spatial similarity for RRR data (DJF, December 2012–February 2016)

Method pair	Correlation	Probability	Similarity
$PV^*$ and *AGP*	$-$ 0.01	$P(PV^*|AGP)$: 0.32**	*0.11* to 0.48**
		$P(AGP | PV^*)$: 0.40**	
$Z^*$ and *AGP*	0.06*	$P(Z^*|AGP)$: 0.78**	0.23 to 0.47**
		$P(AGP | Z^*)$: 0.35**	
$PV^*$ and $Z^*$	0.52**	$P(PV^*|Z^*)$: 0.32**	**0.30** to 0.51**
		$P(Z^* | PV^*)$: 0.90**	

Probability values above 0.7 are bolded. Similarity values are formatted based on relative distributions of values—25th percentile values above (below) 0.29 (0.15) and 75th percentile values above (below) 0.54 (0.39) are bolded (italicized) to denote particularly high or low quantities relative to other values. Statistical significance is denoted by a “*” for $0.01<p<0.05$ and “**” for $p<0.01$

Figure 4 shows the frequency of detected blocking in NP DJF from December 2012 to February 2016, and Table 3 provides the corresponding agreement metrics. Out of the three methods, the one that most closely coincides with the location of the RRR—in terms of a blocking maximum centered off of the West Coast—is the $Z^*$ method. The $PV^*$ method displays an inclination towards contours that co-locate with $Z^*$ contours ($P(Z^*|PV^*)=0.90$), but the maximum is positioned further northwards and there is only a modest correlation of $C(PV^*, Z^*)=0.52$. Due to the $PV^*$ method’s propensity to focus on the ridge peak, $PV^*$ also picks up about half as many instances of ridging (averaged blocking frequency of 9%, $\sim$ 8.28 days per season at its maximum over Alaska) as $Z^*$ (averaged blocking 18%, $\sim$ 16.56 days per season in the location of the $PV^*$ blocking frequency maximum). The *AGP* method has an averaged blocking frequency that about equals $PV^*$ over Alaska, and a high probability of co-occurrence with $Z^*$ ($P(Z^*|AGP)=0.78$), but its maximum during this time is positioned over the Western Pacific, indicating that it is less likely to identify the particular feature that we are interested in identifying here.

**Fig. 5:**
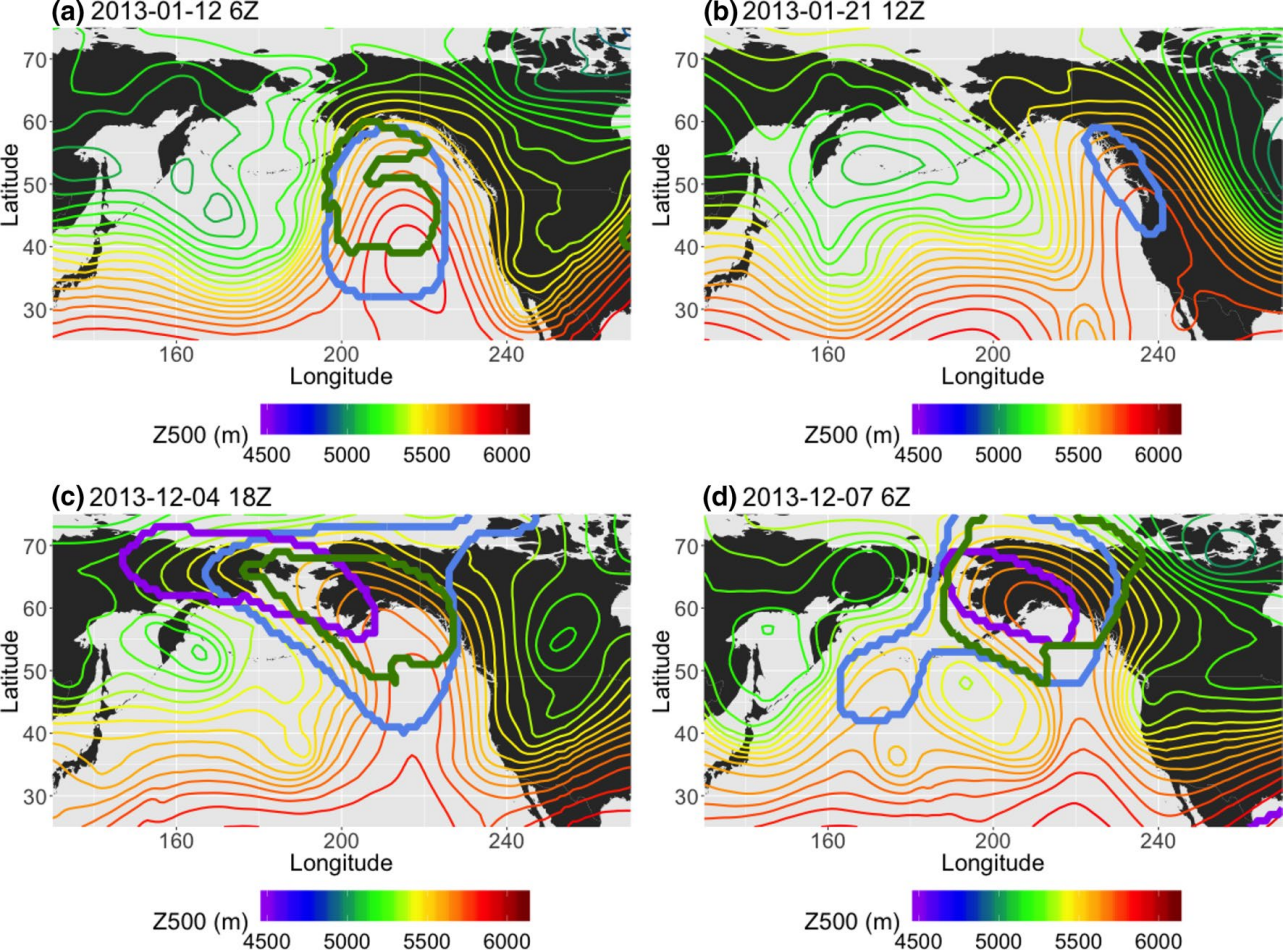
**a**, **b** Examples of ridges that were detected by only the anomaly methods ($Z^*$, blue, and $PV^*$, green) in January 2013. **c**, **d** Examples of ridges that were detected by all three methods (including *AGP*, purple) in December of 2013

The reason for this difference in the average blocking patterns with respect to the RRR becomes apparent in Fig. 5. In each of these examples, the blocks detected by the *AGP*, $Z^*$ and $PV^*$ methods are outlined in purple, blue, and green solid lines, respectively, and the thin contours depict 500 hPa geopotential height in 50 m increments. Many times, the ridge that appears off the North American west coast has a north/south oriented ridge axis with little to no horizontal tilt; therefore, the *GHGS* criterion of the *AGP* method is not fulfilled, as will be discussed in Sect. 4.1. The blocking pattern seen on December 7th, 2013 (Fig. 5d) is one of the exceptions, due to both the slight westward tilt (with increasing latitude) of the ridge axis and the local Z500 maximum at 220E, both of which satisfy $GHGS>0$. However, even in this example, where all three methods detected the feature, they defined the extent of the block differently; comparing the three methods, $S(PV^*,Z^*)=0.56$, $S(PV^*,AGP)=0.38$, and $S(Z^*,AGP)=0.25$. If these detection algorithms were to be used in some sort of predictive capacity, the $Z^*$ method would pick up RRR-like features every time they occurred, but detect many additional features as well. The $PV^*$ method would detect some of the same features as $Z^*$, but it would also miss some instances, as in Fig. 5b, and would not define the extent of the block in the same way. Like $PV^*$, the *AGP* method would also miss some of these features, as well as identifying others that are not relevant here.

If the goal is to detect a ridge configuration similar to that of the RRR, then the results suggest that of the three algorithms discussed in this paper, the $Z^*$ algorithm is the most reliable method to find the ridge, with $PV^*$ acting as a more conservative substitute method. The algorithm design of *AGP* does not deal well with the particular block shape that appears frequently during this time; therefore, the use of *AGP* is not ideal for performing an analysis on future trends in blocking specific to the western United States.

### Blocking climatology by algorithm

**Fig. 6:**
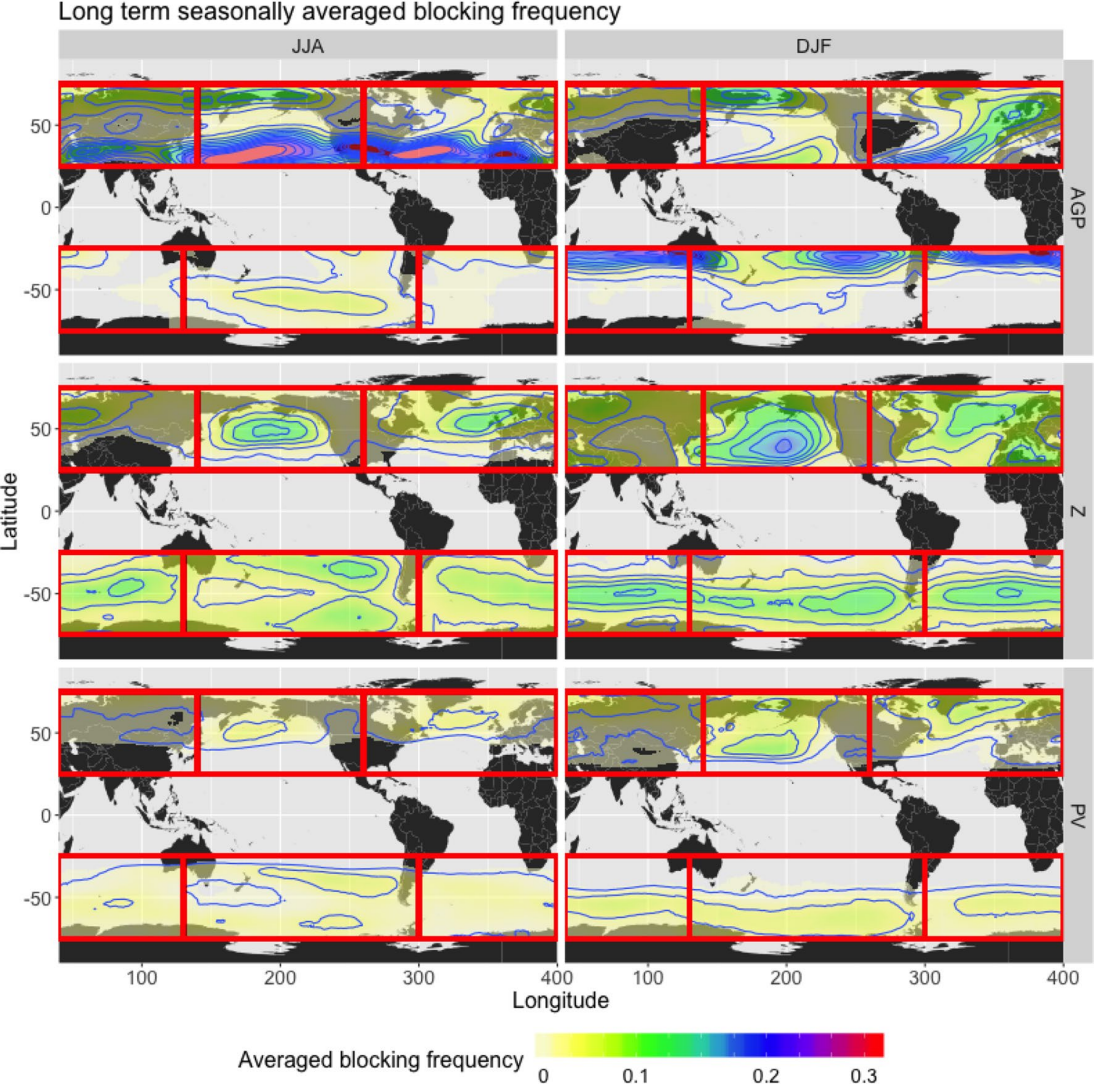
Long term seasonally averaged blocking frequency for (left) JJA and (right) DJF, (top row) *AGP* method, (center row), $Z^*$ method, (bottom row) $PV^*$ method. Frequency values represent the fraction of blocked days per season as averaged over the 39 years of the study, with frequencies here ranging from 0.01 (less than 1 day per season) to 0.40 (about 37 days per season). Contour lines have intervals of 0.03

Figure 6 compares seasonal blocking frequency obtained from applying the three detection methods to the full ERA-Interim reanalysis dataset from 1979 to 2018. Section 3.4 quantifies the amount of agreement between each of the methods, but it is obvious just from viewing the frequency plots that, even using the same time and size thresholds, these algorithms do not agree on the definition of blocking. Each of the three algorithms produce distinct regional and seasonal differences in their overall global blocking climatologies. $Z^*$ is the least discriminating, detecting blocks over the entire extent of each study region (particularly in the winter hemispheres), with maxima of about 19% (18 days per season); in contrast, *AGP* appears more optimized for the NH than the SH, as it detects scarcely any blocks in the SH midlatitudes with the exception of SP, but has distinct maxima of almost 40% (37 days) at the lower latitudes in the summer hemispheres. $PV^*$ has maxima that are similar in location to $Z^*$ maxima, but the lowest overall magnitudes of blocking frequency (maximum values of $\approx 9\%$, or 8 days per season).

These differences are analogous to results reported in previous blocking studies. Certain maxima seen in Fig. 6 are also present in other studies, i.e. the maxima that are centered over Scandinavia and the Aleutian Islands in the NH (Croci-Maspoli et al. 2007, using S04; Dunn-Sigouin et al. 2012, using a hybrid of TM90 and DG83; Cheung et al. 2013, using TM90); and in the SH, the maximum centered in SP (Wiedenmann et al. 2002, using TM90; Parsons et al. 2016, using DG83). The results between papers diverge in terms of the blocking frequency magnitude and the overall extent of blocking, but one could reasonably assume that this was in part due to differences in methodology (choice of variable, anomaly threshold, 1D vs 2D, extent of study region). Here, despite attempts to standardize the detection methodology as much as possible, the three objective detection methods still display differences in magnitude and extent.

One of the most notable differences between methods is seen in the lower latitudes. The 2D blocking indices utilized here select certain blocking patterns that are typically missed by the 1D indices of Tibaldi and Molteni (1990) and others, because 1D methods only identify blocks in the vicinity of a central latitude. Low-latitude blocks are the most obvious example of features that are missed by the 1D methods, and the largest blocking frequencies are all at the lower latitudes. These features will be discussed in further detail in Sect. 4.3; while they tend to be less stationary than other blocking features, they still impair the zonal flow as per the AMS definition and are therefore worth including in the results. *AGP* detects a high percentage of low-latitude blocks in the summer hemispheres (maximum frequency of $\approx 40\%$, or 37 days per season), as well as in the winter hemispheres to a lesser extent. The summer low-latitude blocks are rarely detected by the anomaly methods because deviation from the mean accounts for seasonally high values of their respective variables, but *AGP* has no reference to the mean climatology. However, the anomaly methods do detect low-latitude blocks in winter (particularly in NP DJF), due to seasonally low values of the LTDM.

### Blocking duration, zonal distance traveled, speed, size

This section addresses differences in the block duration, zonal distance traveled, zonal block speed, and block size that emerge from each detection scheme (calculation methods for each of these items is briefly explained in “Appendix B”). It should be noted that all of these characteristics are somewhat intertwined with one another. Smaller detected regions often correlate with shorter durations due to the size constraint, and block speed, being a function of distance and duration, will be skewed by relatively high or low values of either.

**Fig. 7:**
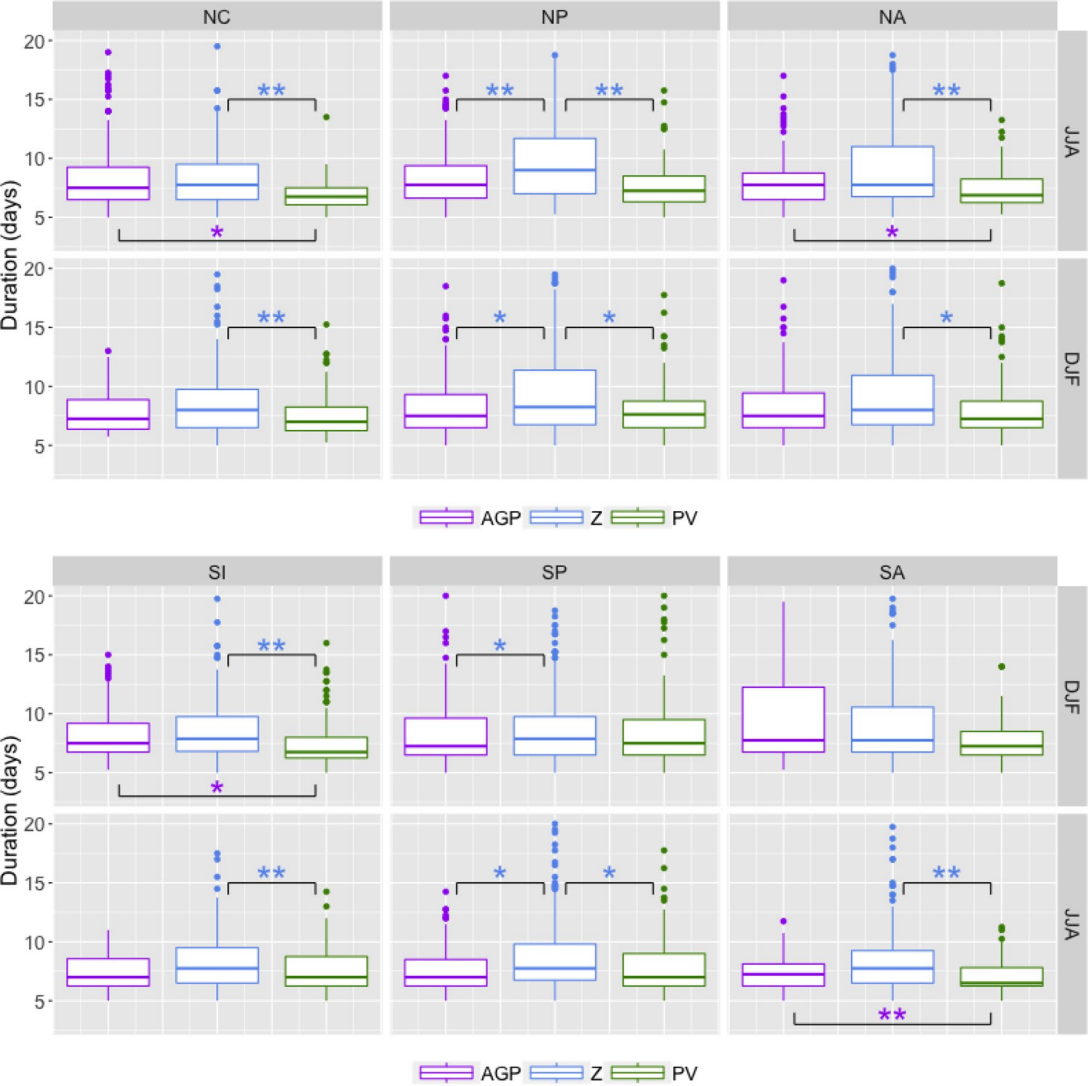
Boxplots of block duration values for NH (top) and SH (bottom), in days. The upper and lower bounds of the box correspond to the 25th and 75th percentile values; the ends of the whiskers correspond to 1.5 times the 25th and 75th percentiles. Dots signify outliers beyond the whiskers. The brackets indicate pairs with statistically significant differences in the median values, with a “*” denoting $0.01<p<0.05$ and a “**” denoting $p<0.01$. The colors of the asterisks indicate which method’s median value is larger (i.e. a purple asterisk indicates that the median value for *AGP* is larger)

**Fig. 8:**
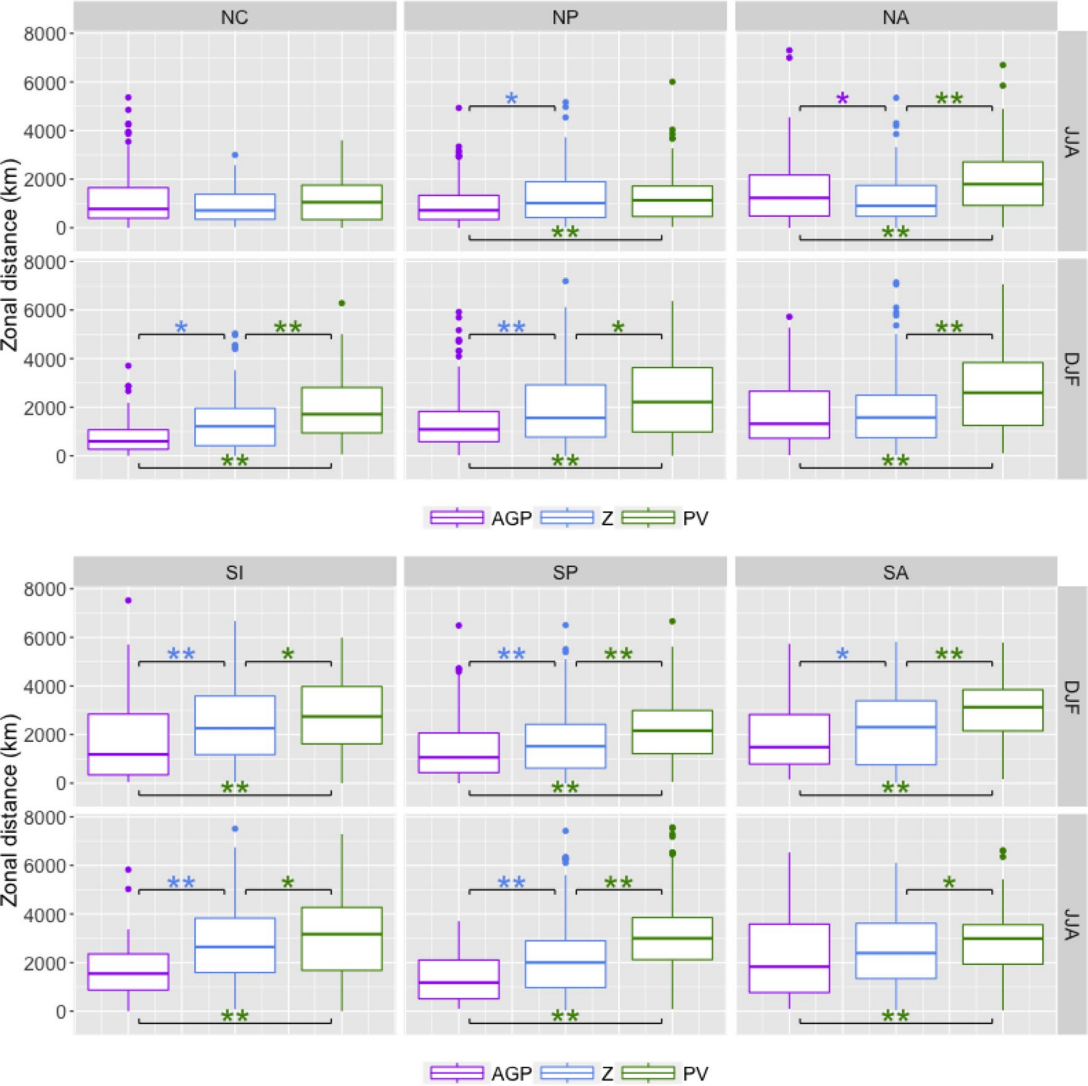
Similar to Fig. 7 except with distance values in km

**Fig. 9:**
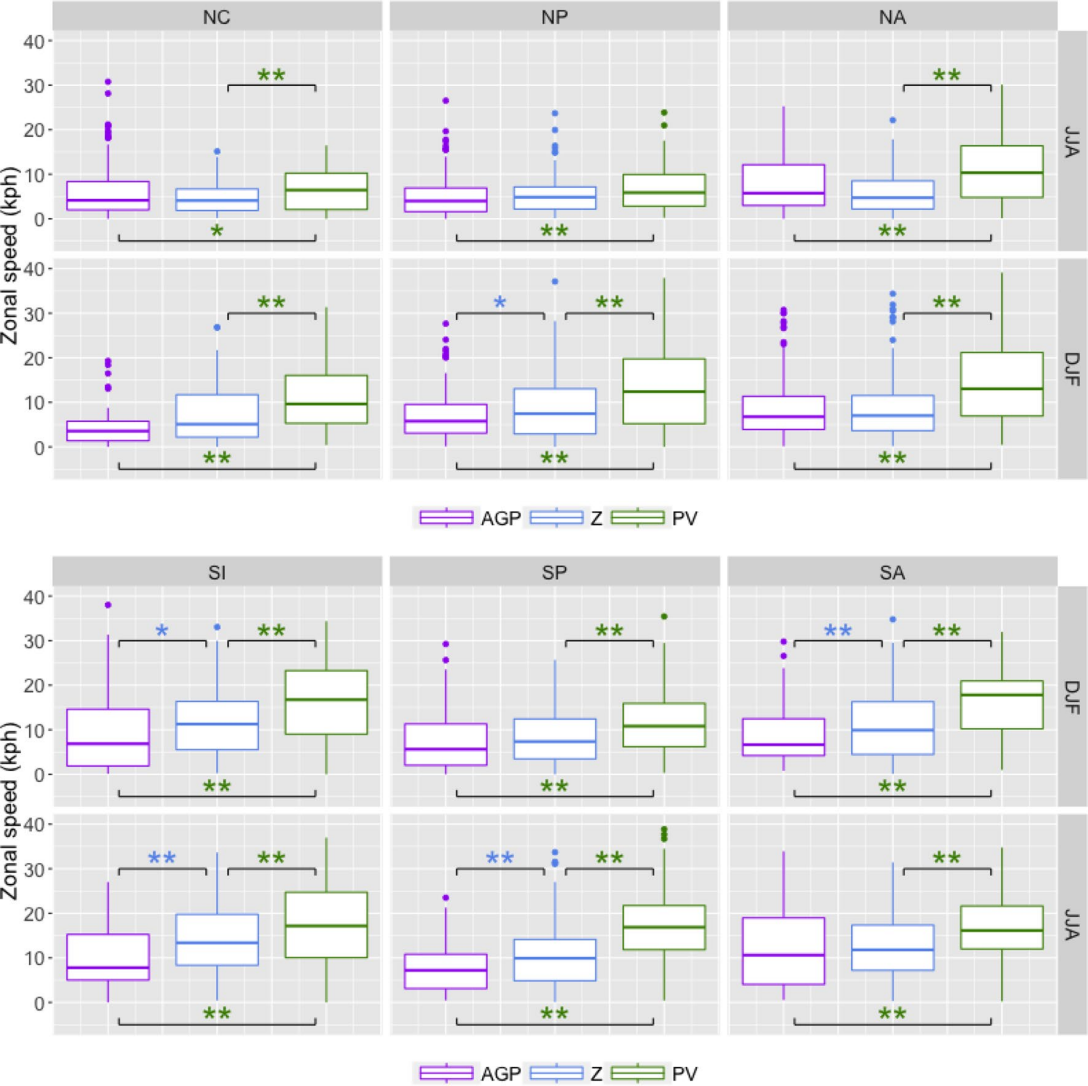
Similar to Fig. 7 except with zonal speed in km/hr

**Fig. 10:**
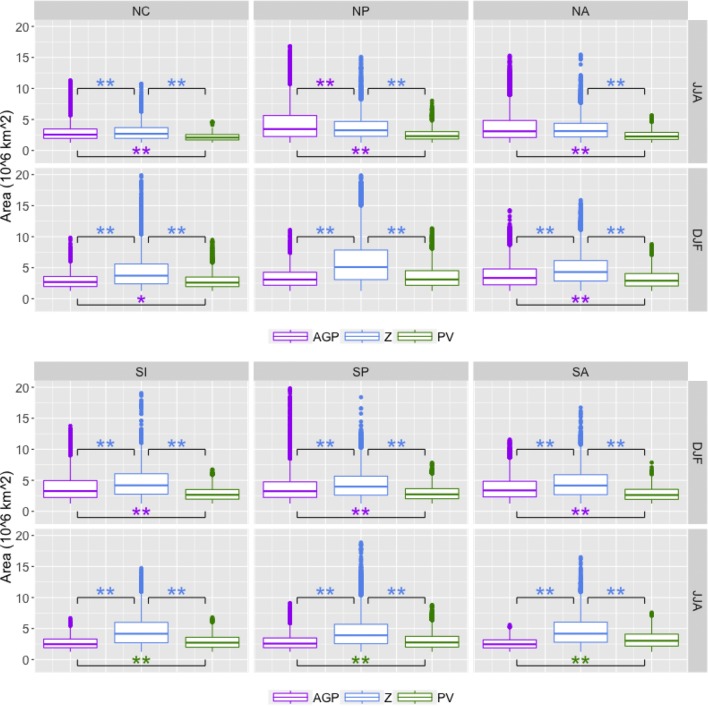
Similar to Fig. 7 except with area in $10^6\,\hbox {km}^2$

Results for each of the methods are presented in Figs. 7, 8, 9 and 10, with the summer season values of the respective hemisphere on the top row and the winter season values on the bottom row. Statistical significance was established by using permutation, as explained in “Appendix E”.

*Block duration* These medians of these results fall within the range of previously presented values (about 6–8 days), such as results from Wiedenmann et al. 2002. The 5-day minimum threshold provides a lower bound to the range of possible 25th percentile values, but the upper bound is quite variable per method, as seen in Fig. 7. $Z^*$-detected blocks have the overall largest durations (as well as most of the largest outlier values), although low-latitude blocks with longer durations values contribute to the upper tail of the *AGP* in the summer hemispheres. $PV^*$ has the smallest overall duration values, but the difference is not always significant, particularly when compared to *AGP*. Smaller duration values are partly an artifact of the size threshold, and $PV^*$-detected regions tend to be smaller and therefore meet the minimum size requirement less often.

*Block zonal distance traveled* As most previous blocking studies determine blocking on a per-gridpoint basis, it is difficult to draw direct comparisons to results here. Figure 8 shows that regions detected by $PV^*$ tend to travel longer distances in the zonal direction (particularly in winter), despite having shorter duration values; statistically significant ($p<0.01$) differences in median values between $PV^*$ and other methods are in the 500–1500 km range. There is a fairly consistent trend in which $PV^*>Z^*>AGP$ in terms of the distribution means, although the differences in the overall distributions are not always significant. It is worth nothing that for the *AGP* method, a larger proportion of cases with distance values greater than 2000 km ($\approx$ 24$^\circ$ longitude) are associated with low-latitude blocking than the other methods; 68% (232 out of 341 cases) of *AGP* blocks with distance values greater than 2000 km were detected equatorward of 40$^\circ$, compared to 4% (29 out of 696 cases) for $PV^*$ and 28% (153 out of 758 cases) for $Z^*$. In general, interquartile ranges of SH distance values are greater than interquartile ranges of NH distance values, and winter is greater than summer.

*Block zonal speed* As with zonal distance, it is difficult to compare these results to previous studies, but Sinclair (1996), which tracks anticyclones in the SH, used a criterion of 3000 km in 5 days (or 25 km/h) as a threshold for limiting tracking to slower-moving features. This threshold was in terms of 2D distance, rather than zonal distance as presented here, but assuming that the trajectory is mainly zonal with slight latitudinal variation, an estimate of 25 km/hr is a reasonable estimate for an upper bound. Speed values, as shown in Fig. 9, tend to follow a similar pattern to distance values, $PV^*$ displaying the largest values and faster speeds in the SH than the NH. $PV^*$-detected regions have the largest zonal speed values due to the combination of shorter duration and longer zonal distance, particularly in the SH; the 25 km/h benchmark from Sinclair is exceeded more often by $PV^*$-detected regions than other methods, and the 75th percentile value is regularly up to 50% larger than that of *AGP* or $Z^*$. The relationship between *AGP* and $Z^*$ consistently shows $AGP<Z^*$ in the SH, but the pattern is less clear in the NH, particularly in NH summer where *AGP* detects a substantial number of low-latitude blocks with large distance and duration values, as noted in the previous sections.

*Block size* It is worth noting that this is the one metric where almost every possible combination of methods, seasons, and regions displayed significant differences between distributions of values. Croci-Maspoli et al. (2007), which uses S04 (but without the size threshold as defined here), presents results in which detected regions in the NH range from 0.5 to $4\times 10^6\,\hbox {km}^2$; Fig. 10 displays the range of block sizes for each method here. The interquartile range for $PV^*$ is largely in agreement with this finding (unsurprising as the algorithm originated in S04), while those of *AGP* and particularly $Z^*$ tend to have larger 75th percentile values and a substantial number of outliers that reach a magnitude of up to $20\times 10^6$ km$^2$, which is 5 times larger than the S04 value. Size is an important consideration here because it can determine whether or not a detected region is rejected due to the threshold constraint. When a blocked feature is detected by both the $PV^*$ and $Z^*$ methods, the larger $Z^*$ contour will frequently both appear earlier and persist longer than the smaller $PV^*$ contour (this is seen in Fig. 16). This is not an artifact of the threshold magnitude, as altering the magnitude of the threshold did not significantly impact the distribution of block size; $PV^*$ blocks on average will be smaller than their $Z^*$ counterparts.

### Intercomparison of blocking algorithms

Tables 4, 5 and 6 provide a summary of the various co-occurrence metrics between the three methods. Each section displays results for the NH and SH as separate subtables, with the summer seasons on top. Correlation and probability tables contain single numbers, as they were calculated as single quantities, but as spatial similarity was calculated for instantaneous fields at each time step containing blocking, there are a range of similarity values, presented here as interquartile ranges similar to the tables in the previous section; there are instances of fields where $S(M1,M2)>0.9$, but these are rare.

**Table 4:** Pearson correlation values between blocking frequencies of each region, as shown in Fig. 6

(a) Northern Hemisphere				
Season	Method pair	NC	NP	NA
JJA	$PV^*$ and *AGP*	$-$ 0.21**	$-$ 0.56**	$-$ 0.46**
	$Z^*$ and *AGP*	$-$ 0.19**	$-$ 0.38**	$-$ 0.49**
	$PV^*$ and $Z^*$	0.51**	0.76**	0.73**
DJF	$PV^*$ and *AGP*	0.62**	*0.01*	$-$ 0.06*
	$Z^*$ and *AGP*	0.26**	$-$ *0.04*	$-$ *0.01*
	$PV^*$ and $Z^*$	0.49**	0.71**	0.45**

(b) Southern Hemisphere				
Season	Method pair	SI	SP	SA
DJF	$PV^*$ and *AGP*	$-$ 0.55**	$-$ 0.54**	$-$ 0.46**
	$Z^*$ and *AGP*	$-$ 0.68**	$-0.64$**	$-$ 0.58**
	$PV^*$ and $Z^*$	0.76**	0.71**	0.79**
JJA	$PV^*$ and *AGP*	$-$ 0.48**	$-$ 0.06*	$-$ 0.59**
	$Z^*$ and *AGP*	$-$ 0.58**	0.09**	$-$ 0.53**
	$PV^*$ and $Z^*$	0.70**	0.44**	0.67**

Magnitudes above (below) 0.7 (0.3) are bolded (italicized) to emphasize patterns of consistently high or low values. Negative values imply an inverse relationship between corresponding gridpoint values. Statistical significance is denoted by a “*” for $0.01<p<0.05$ and “**” for $p<0.01$

*Pearson pattern correlation* The results in Table 4 highlight the clear differences in blocking frequency that will arise given the choice of an anomaly versus a total field (discussed further in Sect. 4.1). Correlation between *AGP* and the other methods is consistently negative in the summer hemispheres due to *AGP* detecting blocks mainly at the lower latitudes, and NC DJF is the only instance of correlation values that are both non-negative and greater than 0.2. In comparison, $C(PV^*, Z^*)$ is almost always the strongest positive correlation value for a particular region or season (with the exception of $C(PV^*, AGP)$ in NC DJF), with particularly strong correlation values in SH summer, NA DJF, SI (both seasons) and NP (both seasons).

**Table 5:** Probability of co-occurrence between instantaneously blocked fields

(a) Northern Hemisphere					
Season	Method pair	Probability	NC	NP	NA
JJA	$PV^*$ and *AGP*	$P(PV^*|AGP)$	*0*.*04*	*0*.*05*	*0*.*04*
		$P(AGP|PV^*)$	0.45	0.46	0.34
	$Z^*$ and *AGP*	$P(Z^*|AGP)$	*0*.*11*	*0*.*18*	*0*.*13*
		$P(AGP|Z^*)$	0.43	0.66	0.44
	$PV^*$ and $Z^*$	$P(PV^*|Z^*)$	*0*.*14*	*0*.*29*	*0*.*24*
		$P(Z^*|PV^*)$	0.48	**0**.**73**	0.60
DJF	$PV^*$ and *AGP*	$P(PV^*|AGP)$	0.38	0.34	*0*.*21*
		$P(AGP|PV^*)$	0.30	*0*.*38*	0.48
	$Z^*$ and *AGP*	$P(Z^*|AGP)$	0.59	0.55	0.52
		$P(AGP|Z^*)$	*0*.*18*	0.41	0.53
	$PV^*$ and $Z^*$	$P(PV^*|Z^*)$	*0*.*24*	0.48	0.31
		$P(Z^*|PV^*)$	0.61	**0**.**73**	0.70

(b) Southern Hemisphere					
Season	Method pair	Probability	SI	SP	SA
DJF	$PV^*$ and *AGP*	$P(PV^*|AGP)$	*0*.*01*	*0*.*04*	*0*.*00*
		$P(AGP|PV^*)$	*0*.*03*	*0*.*11*	*0*.*01*
	$Z^*$ and *AGP*	$P(Z^*|AGP)$	*0*.*09*	*0*.*24*	*0*.*09*
		$P(AGP|Z^*)$	*0*.*16*	0.31	*0*.*14*
	$PV^*$ and $Z^*$	$P(PV^*|Z^*)$	0.40	0.34	0.34
		$P(Z^*|PV^*)$	**0**.**78**	**0**.**73**	**0**.**78**
JJA	$PV^*$ and *AGP*	$P(PV^*|AGP)$	*0*.*13*	*0*.*24*	*0*.*18*
		$P(AGP|PV^*)$	*0*.*04*	*0*.*20*	*0*.*11*
	$Z^*$ and *AGP*	$P(Z^*|AGP)$	0.49	0.66	0.67
		$P(AGP|Z^*)$	*0*.*06*	*0*.*18*	*0*.*14*
	$PV^*$ and $Z^*$	$P(PV^*|Z^*)$	*0*.*26*	0.31	*0*.*28*
		$P(Z^*|PV^*)$	**0**.**72**	**0**.**71**	**0**.**79**

Probability values above (below) 0.7 (0.3) are bolded (italicized) to emphasize high or low values. All probability values are significant at the $p<0.01$ level

*Probability of co-occurrence* The interpretation of the values in Table 5 must take the relative quantities of each method’s detected features into account. For example, it was previously noted that $PV^*$ and $Z^*$ had similar averaged patterns, with some of the highest correlation values in Table 4; however, this does not mean that they are equally likely to predict one another. While $P(Z^*|PV^*)$ is consistently the highest value in a given region, the reverse is not true, since $Z^*$ also detects many other regions that do not coincide with $PV^*$-detected regions. Also, averaged agreement does not necessarily also mean instantaneous agreement; for example, $C(PV^*, AGP)$ is 0.62 in NC DJP but the probabilities of co-occurrence for these two methods are only about 30–38%, while $C(PV^*, Z^*)$ is 0.49 in NC DJF but $P(Z^*|PV^*)=0.61$. In this instance, $PV^*$ and *AGP* detect blocks in similar areas, but the methods are not simultaneously detecting the same features on a per-timestep basis.

**Table 6:** Interquartile ranges of spatial similarity between instantaneously blocked fields

(a) Northern Hemisphere				
	Method pair	NC	NP	NA
JJA	$PV^*$ and *AGP*	0.21–0.46	*0*.*08*–*0*.*38*	0.16–0.43
	$Z^*$ and *AGP*	**0**.**35**–**0**.**58**	*0*.*13*–*0*.*37*	0.19–0.50
	$PV^*$ and $Z^*$	**0**.**33**–**0**.**57**	**0**.**32**–**0**.**54**	**0**.**31**–**0**.**54**
DJF	$PV^*$ and *AGP*	0.27–**0**.**53**	0.15–0.43	*0*.*13*–0.43
	$Z^*$ and *AGP*	**0**.**35**–**0**.**58**	0.19–0.42	0.25–0.50
	$PV^*$ and $Z^*$	**0**.**29**–**0**.**57**	0.25–0.53	0.28–**0**.**54**

(b) Southern Hemisphere				
	Method pair	SI	SP	SA
DJF	$PV^*$ and *AGP*	*0*.*02*–*0*.*21*	*0*.*08*–*0*.*37*	*0*.*02*–*0*.*08*
	$Z^*$ and *AGP*	*0*.*07*–*0*.*23*	0.18–0.39	*0*.*06*–*0*.*24*
	$PV^*$ and $Z^*$	**0**.**36**–**0**.**59**	**0**.**34**–**0**.**62**	**0**.**37**–**0**.**58**
JJA	$PV^*$ and *AGP*	*0*.*05*–*0*.*26*	*0*.*08*–*0*.*31*	*0*.*05*–*0*.*31*
	$Z^*$ and *AGP*	0.24–0.46	0.21–0.43	0.28–0.47
	$PV^*$ and $Z^*$	0.26–0.52	0.23–0.48	0.27–**0**.**54**

The 25th and 75th percentile values are formatted based on the relative distributions of these values— 25th percentile values above (below) 0.29 (0.15) are bolded (italicized), and 75th percentile values above (below) 0.54 (0.39) are bolded (italicized) to denote particularly high or low quantities relative to other values. All ranges of similarity values were significantly different from the generated null distribution

*Spatial similarity* The third measure of agreement between methods, spatial similarity, is highly influenced by the size and location of detected regions relative to one another. Note that the largest 75th percentile value is 0.62, which is unsurprising given the differences in detected block size between methods even if the same feature is detected, as was seen in Fig. 5d. The results in Table 6 provide additional insight to the results from the previous two sections. While $PV^*$ and $Z^*$ again have some of the highest values for similarity relative to other combinations of methods, $S(PV^*,Z^*)$ rarely exceeds 0.6, and more often in the respective summer hemispheres when the $Z^*$ blocks are smaller. Additionally, $S(PV^*,AGP)<S(Z^*,AGP)$ in most instances; this is sometimes due to the relative sizes of the detected contours, but the $PV^*$-detected contour is also sometimes shifted with respect to the *Z*500-based methods (example in Sect. 4.2).

## Meteorological drivers of differences between blocking algorithms

The various metrics displayed in Sect. 3 show that all three of the methods have widely varying definitions of blocks, from the block’s physical characteristics to whether or not a block is actually present at a particular point in time. The RRR case study, in particular, highlights the importance of considering the nature of the region’s flow field and prevailing block type when selecting the appropriate detection method. A few meteorological factors that influence the differences between block detection methods are discussed next.

### Anomaly versus total field Z500-based methods

In Sect. 3.4, the magnitudes of the agreement metrics show that there is a clear distinction between circumstances when blocks are detected by *AGP*, a method based on the total *Z*500 field, as opposed to the other two methods that are based on anomalies with respect to the LTDM. We limit the discussion here to the two methods that are based on *Z*500 in order to reduce other sources of variability.

Both $Z^*$ and *AGP* were created with the purpose of detecting high values of *Z*500, but *AGP* has no reference to the mean climatology; instead, it requires a significant change in *Z*500 over a latitude range (30$^\circ$) that equates to more than half of each study region’s latitude range (50$^\circ$). The difference in block detection is most evident in the SH, where there is a much stronger zonal flow component than in the NH. This stronger zonal flow implies that the requirements of the *AGP* method are fulfilled far less often in the SH midlatitudes, since there is not sufficient distortion of the flow field; *AGP*-detected blocks in the SH midlatitudes are often co-located with lows (dipole or omega blocks) or have a tilt in their north-south axis, since this guarantees that there is a sufficiently large *Z*500 gradient. In contrast, $Z^*$ is much less discriminating; it detects a high number of blocks in the SH because the $\overline{Z500}$ field has a strong meridional gradient in addition to its mainly zonal flow, meaning that even fairly shallow ridges in the *Z*500 field are more likely to satisfy $Z^*$ anomaly thresholds.

**Fig. 11:**
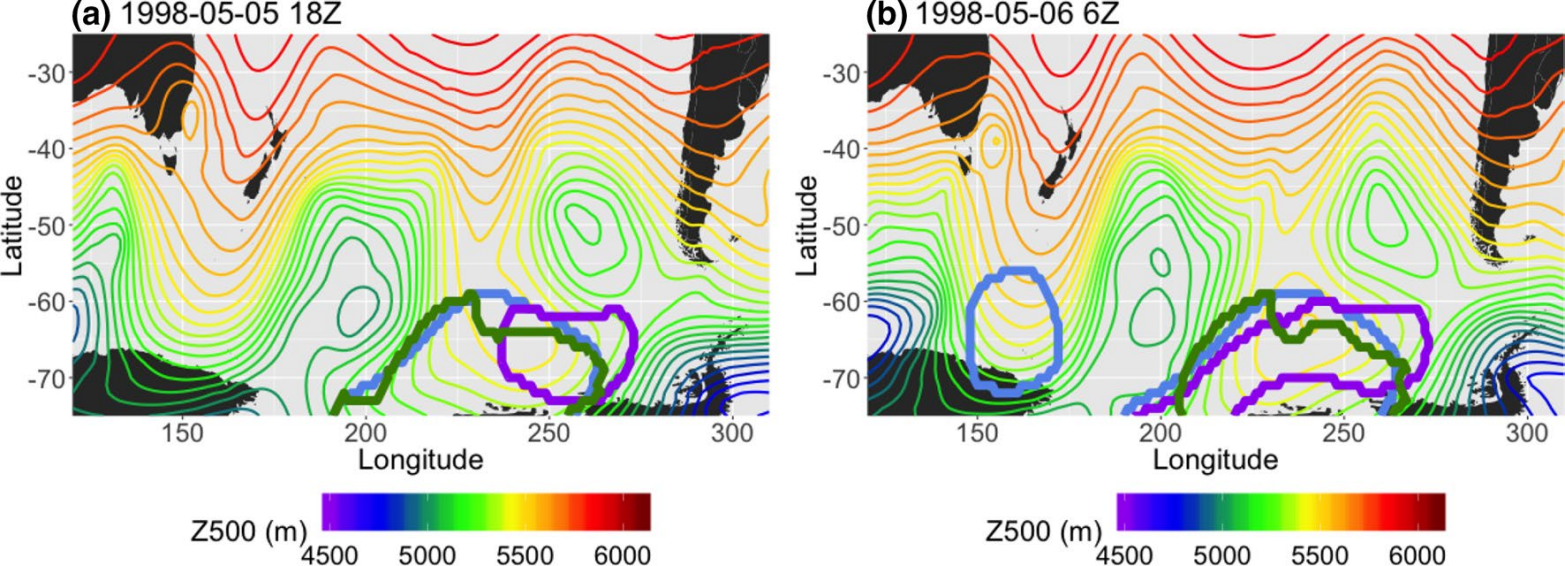
Example, 12 h apart in 1995 MAM, of instances in which there is **a** less and **b** more agreement between the *AGP* method (purple) and the two anomaly methods (blue and green) in SP

These points are illustrated in Fig. 11, which shows a snapshot of an omega block in SP on May 5th 18Z 1998 (left) and 12 hours later (right). At the earlier time, the two anomaly methods produce clusters that are centered over the ridge ($S(PV^*, Z^*)=0.78$), but the *AGP* method only picks out an area centered polewards of the lower geopotential heights to the east of the ridge (before the size constraint was applied, there was also a detected region in the vicinity of 190E,65S); $S(PV^*, AGP)=0.24$ and $S(Z^*, AGP)=0.26$. Later, the intensification of the high in the 60S–70S latitudes produces the necessary height gradient to satisfy the criteria for *AGP*; $S(PV^*, AGP)=0.46$ and $S(Z^*, AGP)=0.58$.

### $PV^*$ links to shear and vorticity

The $PV^*$ method picks out regions with PV that are highly anomalous with respect to the climatological mean in the upper troposphere. Most often, these regions are areas with particularly pronounced anticyclonic circulation in the *Z*500 field, such as dipoles or omega blocks. Since anomalous vorticity and anomalous highs are linked, the two anomaly methods are often very similar in terms of the location of the detected block, even if the size of the detected cluster of grid points is not always the same. However, the EPV field can be influenced by phenomena such as vertical shear (first two terms in Eq. 8) or locally strong winds; it is a factor to consider in flow that does not have the necessary meridional component to satisfy points 1 and 2 of the AMS definition of blocking. For example, a jet streak embedded in otherwise zonal flow will lead to mistaken identification of a region as blocked even though there is neither persistent obstruction nor pronounced meridional flow. The $PV^*$ method tends to detect gridpoints at higher latitudes than the other two methods during each respective hemisphere’s summer, and over a wider range of latitudes in the winter; this corresponds roughly to the location of the 500 hPa jet, which shifts from polewards of 45$^\circ$ latitude in summer to 40$^\circ$ latitude and below in the wintertime. Therefore, the $PV^*$ method is somewhat linked to the presence of the jet stream and embedded jet streaks.

**Fig. 12:**
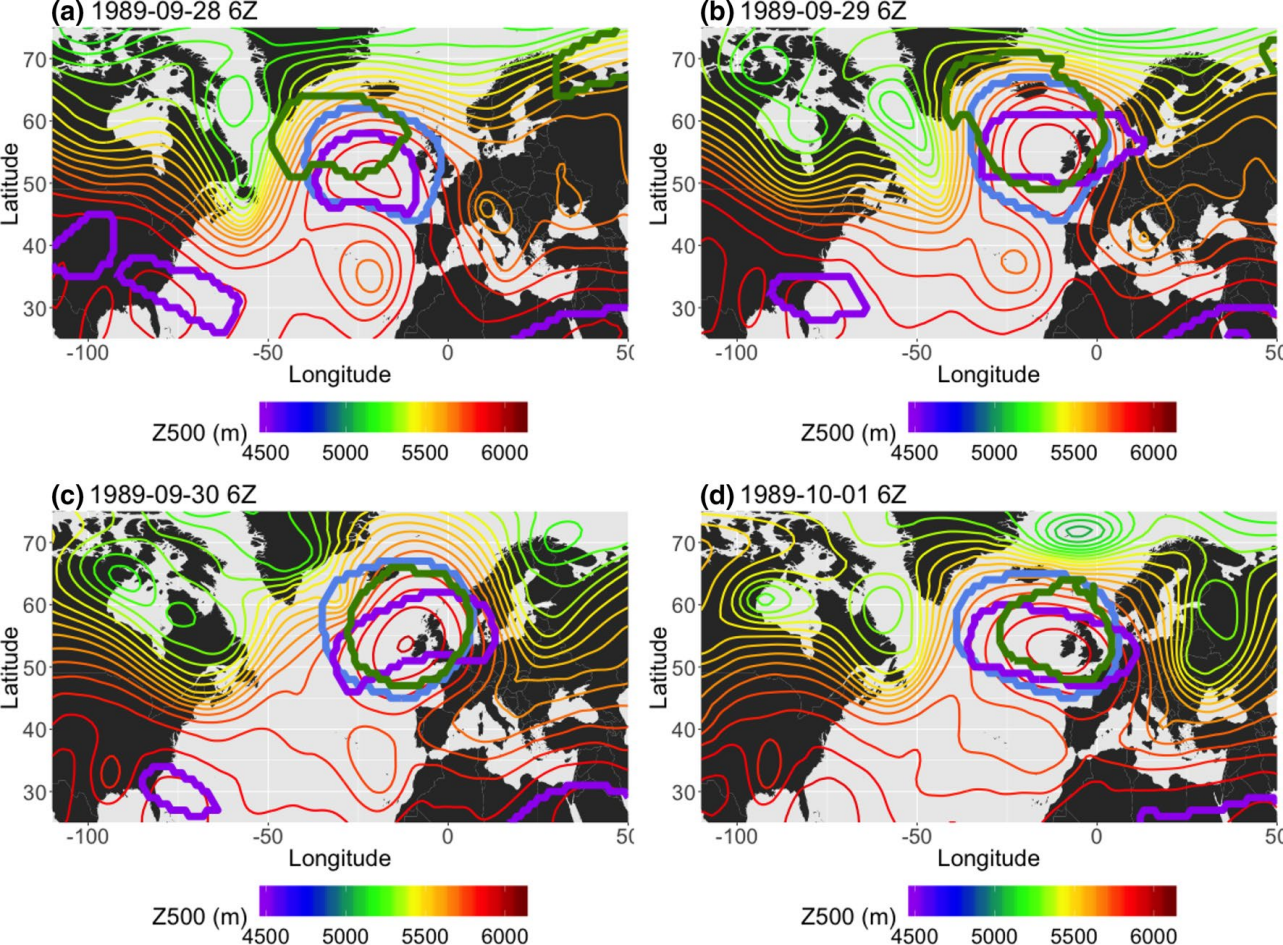
Example, in 24-h increments, of omega block detection in 1989 NA SON. The $PV^*$ method is denoted by the green contour, the $Z^*$ method is blue, and the *AGP* method is purple

**Fig. 13:**
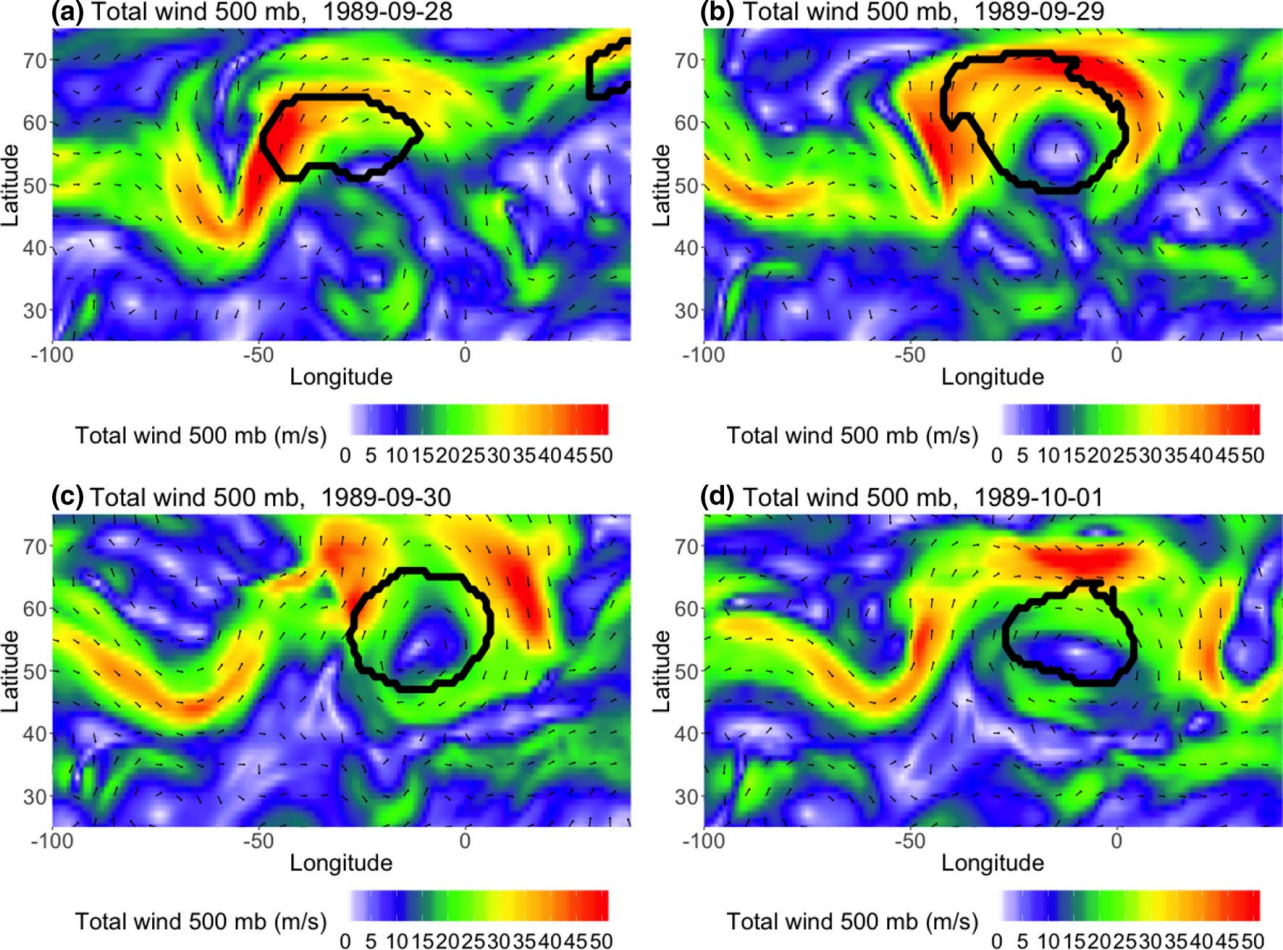
500 hPa vector wind field corresponding to previous figure (September 28th–October 1st), showing location of jet streaks. Wind speeds upwards of 45 m/s are visualized as the red areas. The thick black contour corresponds to the blocked region detected by $PV^*$

Figure 12 demonstrates a case in which a $PV^*$-detected cluster is shifted relative to clusters detected by the other two methods, despite all of the methods identifying the same block. The 4-panel figure shows an omega block that is detected by all three methods in 1989 NA SON; however, in Fig. 12a, the $Z^*$ and *AGP* contours both center on the high that is the top half of the block ($S(Z^*, AGP)=0.56$), while the $PV^*$ contour is shifted westwards ($S(PV^*, Z^*)=0.29$, $S(PV^*, AGP)=0.18$). Daily composites of the corresponding total wind (Fig. 13) show that, on September 28th 6Z, 1989 (Fig. 13a), there was a jet streak on the upstream side of the high pressure feature which is being detected by the three algorithms. The vorticity ($-g\zeta \frac{\partial \theta }{\partial p}$ in Eq. 8) associated with the anticyclonic curvature of the high is strongly negative at this time, and further enhanced by vertical shear in the jet streak region. The combination of these factors leads to the westward extension of the $PV^*$-detected cluster relative to the other two clusters. As time progresses (Fig. 13b), a portion of the $PV^*$-detected contour continues to track that jet streak, which coincides with strongly negative vorticity, until it reaches the downstream side of the omega block (Fig. 13c). In Fig. 13d, the next jet streak has reached a higher latitude, where $\overline{PV}$ is larger and there is positive vorticity associated with the low. The anomaly is no longer exceeding the threshold; therefore, the $PV^*$ cluster separates from the jet streak and is in better agreement with the other two methods ($S(PV^*, Z^*)=0.52$, $S(PV^*, AGP)=0.54$).

### Low-latitude blocking and flow impairment

Low-latitude blocking with respect to NH summer and the *AGP* method is discussed in Davini et al. (2012); their paper questions whether these features are correctly characterized as blocks, since they are linked to poleward displacement of subtropical easterlies and are less intense and persistent than those at higher latitudes. Low-latitude blocking detection is present in all three methods: the *AGP* method has relative maxima in the averaged blocking patterns in the respective summer hemispheres, and all three methods find low-latitude blocks in the respective winter hemispheres ($PV^*$ and $Z^*$ have distinct maxima in the lower latitudes of NP DJF). Many of these low-latitude features are nearly stationary (block speed averages 2–12 km/h in both hemispheres) and persistent (block duration averages 6–12 days in both hemispheres). We present two cases of low-latitude blocking here: one summer case, and one winter case.

**Fig. 14:**
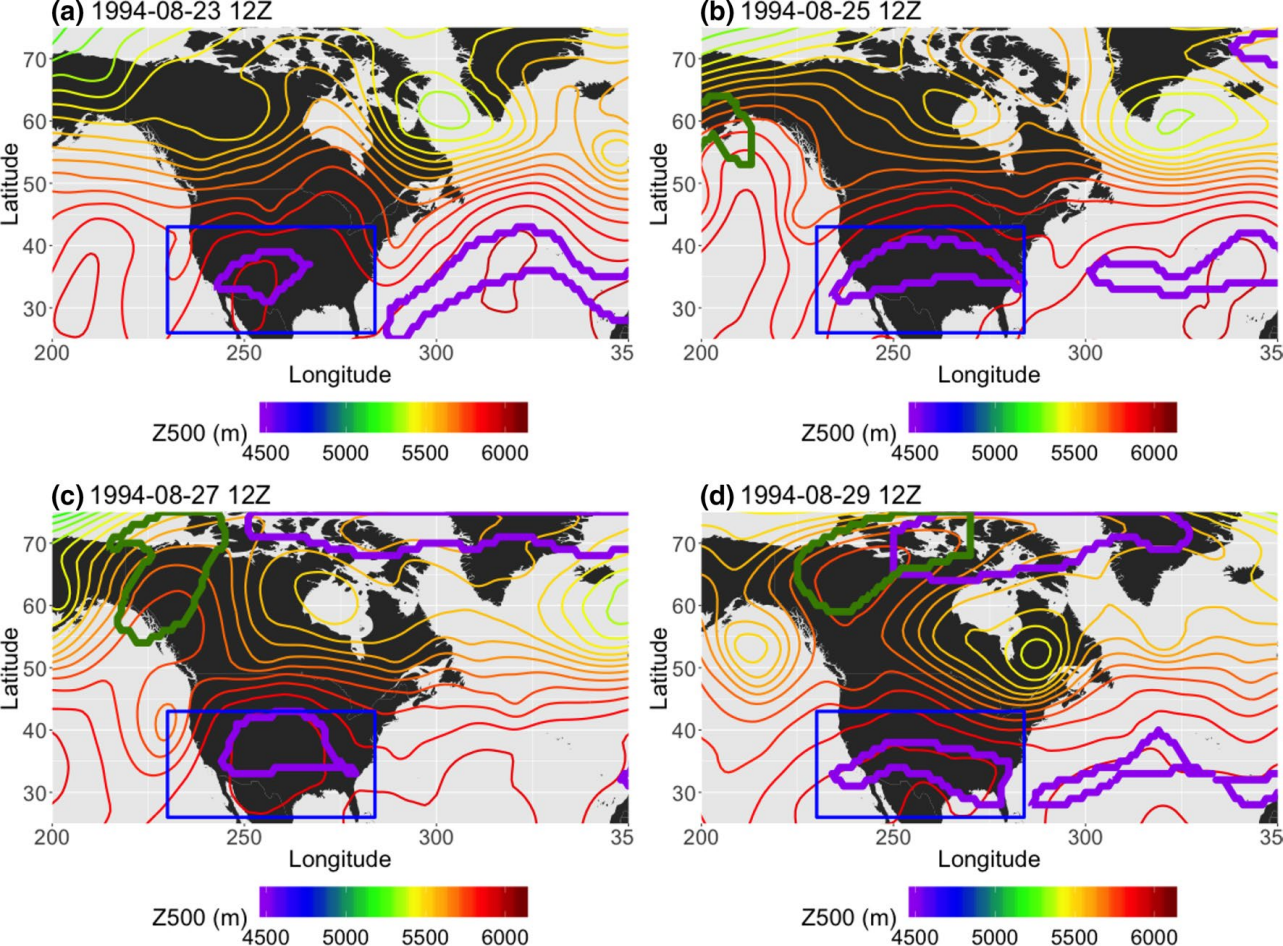
Example, in 48-h increments, from NP JJA 2014, of a low-latitude block detected by the *AGP* (the other two methods do not detect a block here). Thin contours are Z500 in 50m intervals, and the thick purple contour denotes the detected feature. The blue box spans [230E–234W, 25N–43N] and outlines the extent of the detected block

**Fig. 15:**
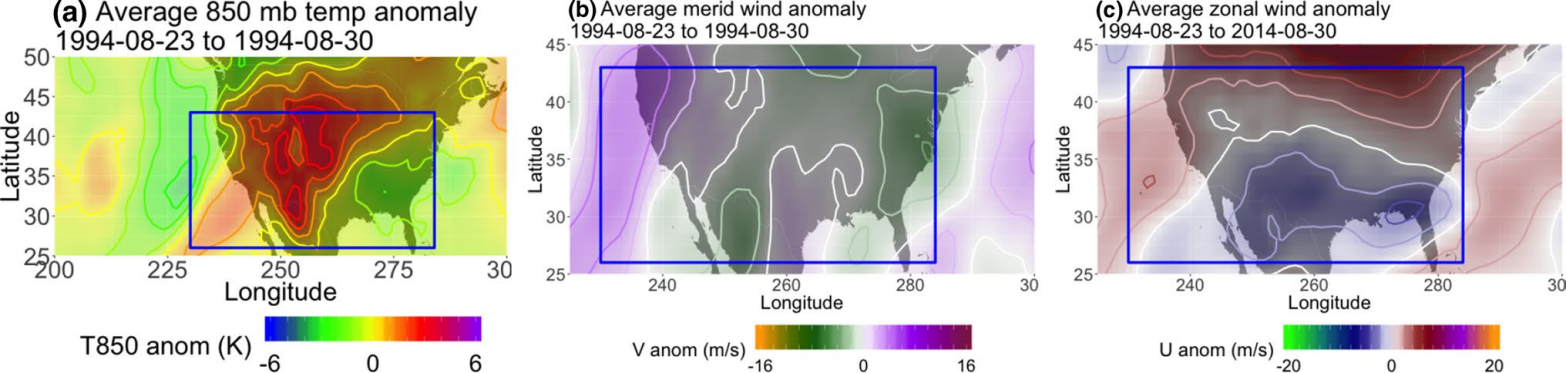
Averaged **a** 850 hPa temperature, **b** 500 hPa meridional, and **c** 500 hPa zonal wind anomalies for June 8th–17th 1984. The temperature contour intervals are 1K and the wind contour intervals are 2 m/s. The blue box corresponds to the one seen in Fig. 14

Figure 14 shows an example of one of the more stationary low-latitude *AGP*-detected regions in JJA, a persistent ridge over the central United States in 1994 that lasted from August 23rd 12Z to August 30th 0Z and had an average zonal speed of 0.74 km/h over 7.75 days. The other two methods did not detect this feature, although the $PV^*$ method detected a second feature at the higher latitudes of Fig. 14b–d. Averaged over the detected block’s lifespan, the 850 hPa temperature anomaly ($T_a$) in the vicinity of the detected block is approximately + 4K, the 500 hPa meridional wind anomaly ($v_a$) is approximately $\pm\, 5$ m/s on either side of the block, and the 500 hPa zonal wind anomaly ($u_a$) is up to −5 m/s in the blocked flow region (see Fig. 15). The size and shape of the *AGP*-detected region fluctuates from panel to panel as the height gradient changes, a trait that is more common among *AGP*-detected regions (particularly at the lower latitudes) than their $Z^*$ or $PV^*$ counterparts. The resultant averaged wind anomalies are somewhat weaker than higher latitude cases, but the flow is indeed being consistently diverted over the course of the ridge’s existence, and therefore satisfies the first point of the AMS definition (as well as somewhat satisfying the second point of meridional flow).

**Fig. 16:**
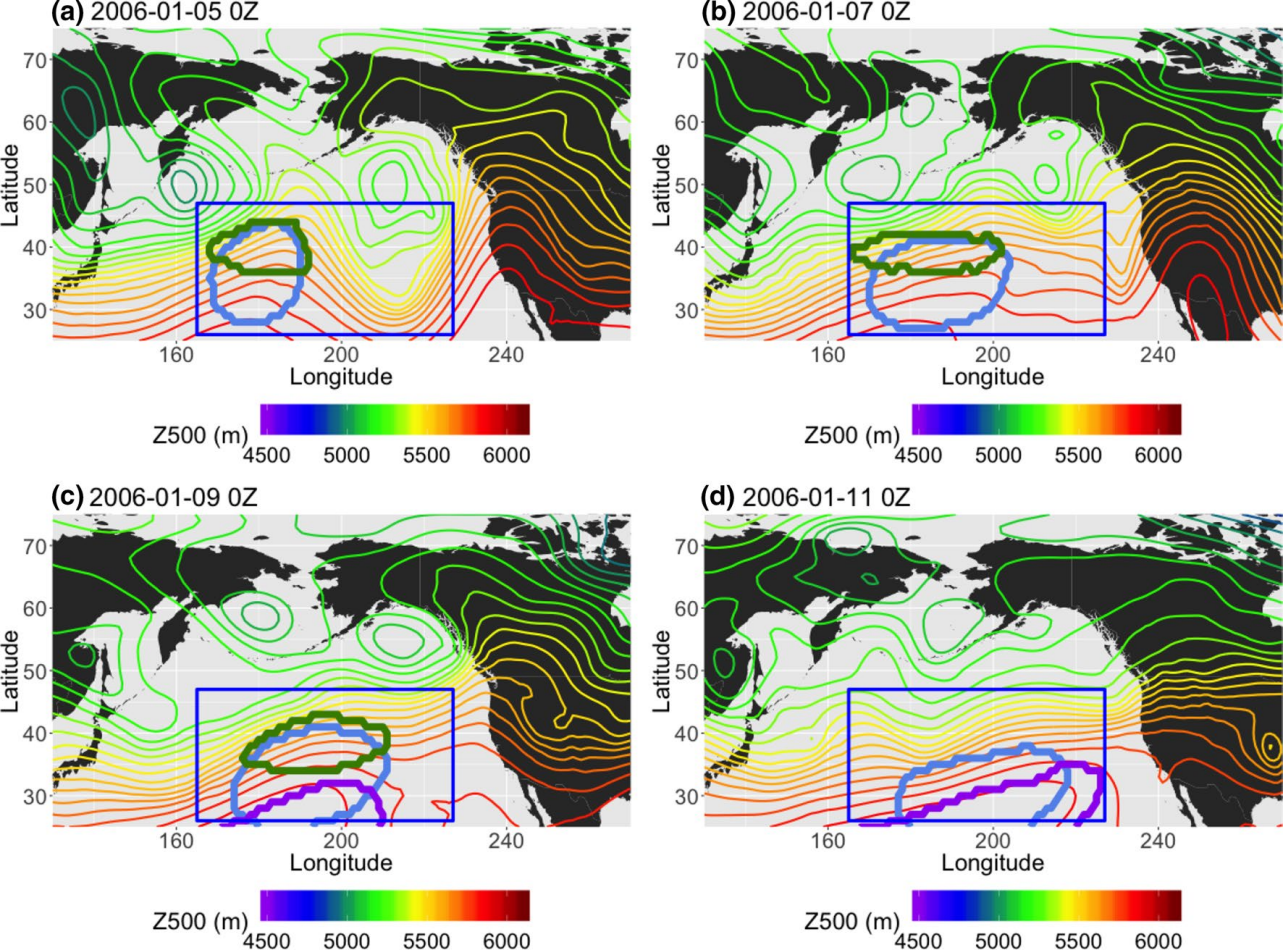
Example, in 24-h increments, from NP DJF 2006, of a low-latitude block detected by the *AGP* (purple), $Z^*$ (blue), and $PV^*$ (green) methods. The blue box spans [165E–227E, 26N–47N] and outlines the extent of the detected block

**Fig. 17:**
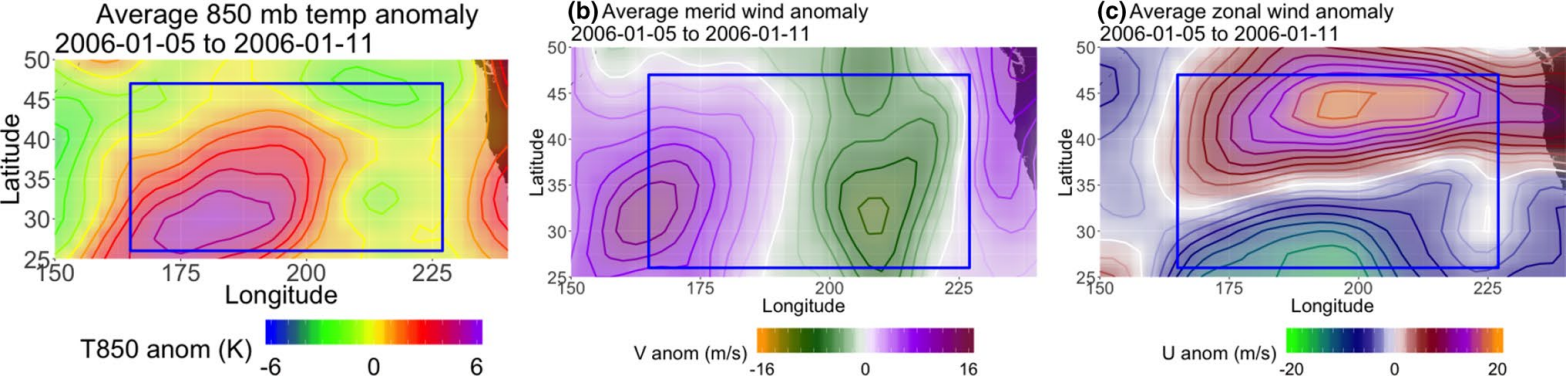
Averaged **a** 850 hPa temperature, **b** 500 hPa meridional, and **c** 500 hPa zonal wind anomalies for January 6th–11th 2006. The temperature contour intervals are 1K and the wind contour intervals are 2 m/s. The blue box corresponds to the one seen in Fig. 16

**Fig. 18:**
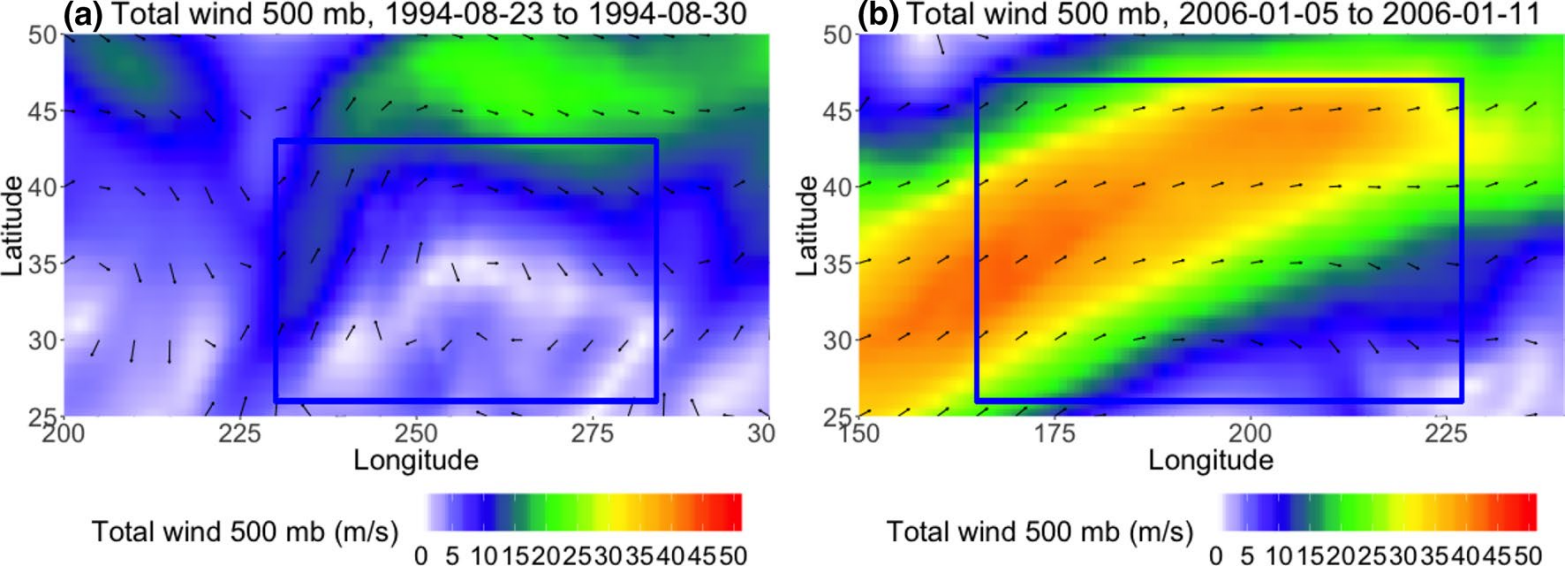
500 hPa total wind fields and vectors for **a** the JJA blocking case in Fig. 14 and **b** the DJF blocking case in Fig. 16. The vectors indicate the wind direction, and the colors indicate the wind magnitude

In contrast, Fig. 16 shows a low-latitude case in NP DJF in which all of the methods identify a block in the midst of flow that is not sufficiently diverted or slowed. This example shows January 5th 0Z to January 11th 0Z, 2006, where all three methods detected a relatively shallow ridge that evolves into mostly zonal flow by the end of the detected block’s lifespan, although the *AGP* method only detects the ridging at the lower edge of the study region during the last few days (Fig. 16c, d) and the $PV^*$ method disappears in Figure 16d. The $PV^*$-detected region has an average zonal speed of 12 km/h over 8 days and the $Z^*$-detected region has an average zonal speed of 10 km/hr over 12.5 days (the difference is mainly due to the longer lifespan of the $Z^*$-detected region). The *AGP*-detected region has an average zonal speed of 7.86 km/h over 5.25 days (and it mainly tracks the ridge after the other two methods have stopped following it, outside of the time window examined here). $u_a$ is $-16$ m/s in bottom half of the blocked flow region outlined in Fig. 17, but northwards of 35N, $u_a$ has a maximum magnitude of 20 m/s. $v_a$ is $\pm 10$ m/s on either side of the block and $T_a$ is + 5K within the blocked region, both of which are larger anomalies than the JJA case. However, one must always approach anomalies with caution; the Z500 field in Fig. 16 does not indicate that the zonal flow has been reduced at later times. Indeed, composites of the total wind fields in Fig. 18 show that while the JJA case has reduced wind speeds (maximum 5 m/s in the blocked region, compared to 20 m/s outside the impaired region), the DJF case has wind speeds of up to 35 m/s in the northern portion of the “blocked” region, which also coincides with the positive $u_a$ value in the northern half of the outlined region in Fig. 17. In the case of $Z^*$, this mistaken identification can be attributed to a fairly shallow ridge in an area with a strong meridional gradient (as in Sect. 4.1). In the case of $PV^*$, vorticity values were only slightly negative or close to 0, but when considering *VPV* compared to $\overline{VPV}$, sufficiently anomalous for $VPV^*$ to surpass the local threshold value of 1.2 PVU. In both instances of *AGP*-detected blocking, the presence of a sustained ridge was enough to qualify as blocked, regardless of other criteria.

These examples suggest that not all low-latitude blocking should be discounted, at least according to certain metrics; thus, applying blocking algorithms over a range of latitudes rather than a single central latitude will produce a more complete climatology of blocking hotspots. A number of detected low-latitude blocks will partially meet, or fail to meet the AMS flow diversion criteria; the first case detected a region of impaired flow, albeit a fairly weak one; and the second case did not produce demonstrably impaired flow at all. However, algorithm “failures” are not unique to the lower latitudes; therefore, rather than restricting analysis to a limited range of latitudes, future research should consider algorithm limitations, which have been mentioned in previous sections, and make use of additional diagnostic metrics to filter out non-blocked flow.

## Conclusions

This study examines the blocking climatologies of six regions over summer and winter seasons, and highlights some of the block characteristics from three detection methods, which can be seen in Table 7. Maximum blocking frequency ranged from 9% ($PV^*$) to 40% (*AGP*), with the locations of maximum blocking differing between *AGP* and the anomaly methods. Some methods, such as the $PV^*$ method, are quite conservative, showing comparatively smaller frequencies and detecting smaller clusters; while others, such as the $Z^*$ method, are less discriminating with respect to regions that are defined as blocked, displaying both higher frequencies and larger clusters.

**Table 7:** Summary of notable blocking frequency distribution and block characteristics

	*AGP*	$Z^*$	$PV^*$
Flow field sensitivity	Equatorward low	Strong high	Strong curvature
	Strong poleward *Z*500 gradient	Strong *Z*500 meridional gradient	Strong winds, vertical shear
	Subtropical high pressure		
Favored block types	Omega block or ridge with meridional axis tilt	All kinds (with sufficiently large $Z500^*$)	All kinds (with sufficiently large $VPV^*$)
	Cutoff low		
Spatial distribution of frequency	NH: Higher latitudes, Atlantic Ocean basin (and some East Pacific), summer low latitudes	Blocks detected over full range of study regions	Higher latitudes in summer seasons, wider range of latitudes in winter seasons
	SH: summer low latitudes, SP midlatitudes		
Location (frequency) of maximum	NH JJA equatorwards of 45$^\circ$ (40%)	NP DJF equatorwards of 45$^\circ$ (19%)	NP DJF equatorwards of 45$^\circ$ (9%)
Notable features	Less similar to other two methods, highest frequency of low-latitude blocking	Highest frequency of SH blocking	Lowest overall frequency magnitude between methods
Duration	$\approx$ 6.5–10 days	$\approx$ 6.5–11 days	Shortest overall between methods ($\approx 6.25{-}9$ days)
Zonal distance traveled	$\approx$ 600–2500 km; shortest between methods in NH winter and SH (both seasons)	$\approx 800{-}3100$ km	Longest between methods ($\approx 1200{-}3700\,\hbox {km}$) with exception of NH summer ($\approx 600{-}2100\,\hbox {km}$)
Zonal speed	$\approx$ 3–12 km/h; slowest between methods in NH winter and SH (both seasons)	$\approx 4{-}13\,\hbox {km/h}$	Fastest overall between methods ($\approx 6.5{-}18$ km/h)
Size	Smallest between methods in SH winter ($\approx 1.5{-}2.8\times 10^6\,\hbox {km}^2$); $\approx 1.8{-}4.2\times 10^6\,\hbox {km}^2$ otherwise	Largest overall ($2.2{-}5.2\times 10^6\,\hbox {km}^2)$	Smallest between methods ($1.6{-}3.4\times 10^6\,\hbox {km}^2$) except SH winter

**Table 8:** Summary of notable observations for intercomparison of objective detection methods

	$PV^*$ and *AGP*	$Z^*$ and *AGP*	$PV^*$ and $Z^*$
Correlation	Weak to moderate negative correlation	Weak to moderate negative correlation	Moderate to strong positive correlation
Probability of co-occurrence	Low to moderate for both $P(PV^*|AGP)$ and $P(AGP|PV^*)$; particularly low in SH	Moderate $P(Z^*|AGP)$ in winter, $P(AGP|Z^*)$ in NH summer; low otherwise	Moderate to high $P(Z^*|PV^*)$ in all regions
Spatial similarity	Lowest ranges of similarity values compared to other method pairs	$S(Z^*,AGP)>S(PV^*,AGP)$	Highest range of similarity values compared to other method pairs in almost all regions and seasons

The intercomparison of the results, as summarized in Table 8, raises some points that should be considered in future blocking studies that use objective detection algorithms. Previous blocking studies often present averaged results, rather than examining them on a per-block basis, but high averaged pattern correlation does not imply that two methods will simultaneously detect the same features; furthermore, even when all three methods detect the same feature, they do not necessarily define the block using the same cluster of gridpoints. The *AGP* and anomaly methods had a much lower degree of agreement than that between $PV^*$ and $Z^*$, in both the averaged and instantaneous sense. However, even the agreement between $PV^*$ and $Z^*$ is affected by the relative sizes and placement of the detected clusters, and the RRR case study demonstrates that the block configuration is an important element to “successful” detection of a block. This has implications to studies which attempt to link blocking to extreme weather, because attempts to correlate the location of the block with the location of extremes will produce different results based on the chosen algorithm.

The ideal blocking detection algorithm (one that detects all features that satisfy the AMS definition) likely requires elements from multiple detection algorithms (such as Dunn-Sigouin et al. (2012) or Barriopedro et al. (2010), which combine elements of TM90 and DG83), as well as measurements of metrics such as block intensity (developed by Wiedenmann et al. 2002) and flow diversion. Additionally, such an algorithm would need to take seasonal and regional differences of the flow field into account. The results show that there remains a significant discrepancy between published methods with regards to how the AMS definition is interpreted, from the calculated blocking frequencies to the average size and speed of the detected features. Many of the detected regions are persistent in the sense that they are relatively nonstationary for at least 5 days, but the resultant changes to wind speed and temperature are inconsistent. Blocks are a continuum of forms, rather than clearly delineated idealized shapes; and each method is optimized for detecting certain kinds of features under certain kinds of climatological conditions.

This study has produced a comprehensive summary of the factors that influence block detection by three different algorithms, and noted the relative strengths and weaknesses of these methods. Additionally, we have outlined a number of metrics for both assessing individual methods and comparing the results between methods. As part of this study, a publicly-available software package has been developed that can be used for automated block detection and tracking in arbitrary global climate datasets. Objective algorithms show promise for analyzing current and future trends, given their applicability to extremely large volumes of high resolution data. Using results from historical models can provide a confidence bound on anticipated changes in blocking characteristics in future climate simulations, but careful consideration of algorithm biases should be factored into the analysis.

## Code availability

All of the analysis for this paper was performed using the TempestExtremes software package, which is available for use under the Lesser GNU Public License (LGPL). The blocking and statistics code can be obtained from GitHub at https://github.com/ClimateGlobalChange/tempestextremes.

## Appendix A: Vertically averaged potential vorticity

In its simplest form, Ertel’s potential vorticity (EPV) is written as

7 $$\begin{aligned} EPV = \frac{\zeta _a\cdot \nabla \theta }{\rho } \end{aligned}$$

Assuming hydrostatic balance in order to eliminate density, (7) can be written as

8 $$\begin{aligned} EPV=g\left( \frac{1}{r\cos \phi }\frac{\partial v}{\partial p}\frac{\partial \theta }{\partial \lambda }-\frac{1}{r}\frac{\partial u}{\partial p}\frac{\partial \theta }{\partial \phi }-(\zeta +f)\frac{\partial \theta }{\partial p}\right) \end{aligned}$$

in which *g* is the gravitational constant, *r* is the Earth’s radius, *u* and *v* are the zonal and meridional wind components respectively, $\theta$ is potential temperature, and $\zeta + f$ is absolute vorticity.

The form presented in Eq. 8 is in spherical coordinates ($\lambda , \phi$) in the horizontal direction and pressure level coordinates (*p*) in the vertical.

For S04, the EPV is vertically averaged over the 150–500 hPa layer using the integral method,

9 $$\begin{aligned} VPV=\frac{1}{(p_1-p_2)}\int _{p_1}^{p_2} EPV(p) dp \end{aligned}$$

where $p_1=1.5\times 10^4$ Pa and $p_2=5\times 10^4$ Pa, respectively.

## Appendix B: Blocking events and the StitchBlobs software

All of code used to produce these results are included in the TempestExtremes software package (Ullrich and Zarzycki 2017). The algorithm for the *AGP* method is contained to a single binary, BlockingGHG, while the calculations for $PV^*$ and $Z^*$ involve a multi-step process which is outlined in Sect. 2.2.4 and Fig. 1.

The StitchBlobs binary takes the outputs from the desired algorithm and applies the spatiotemporal constraints, and the corresponding BlobStats binary produced summary text files which include per-feature information about:

- Minimum/maximum latitude and longitude coordinates
- coordinates of block centroid
- block area (in terms of fractional area)

This information is mainly used in Sect. 3.3, and the quantities are defined as follows:

- *Block duration* the number of time steps for which block is present, multiplied by the time resolution.
- *Block zonal distance* The difference between the start and end longitudes, converted to km.
- *Block zonal speed* Distance divided by duration.
- *Block size* Area in steradians multiplied by the Earth’s radius squared, $A_{\text{km}} = A_{\text{str}} (6371)^2\,\hbox {km}^2$.

A previous attempt to average speeds calculated over each 6 hour period was discarded because the center coordinate would occasionally “jump” in between subsequent time steps due to a change in the detected block’s size—usually caused by merging of distinct features—and a subsequent shift in the centroid. This “jump” led to large zonal distance values and, consequently, artificially high block speed values. The method used here (distance from start to finish, divided by duration) has its own drawbacks, as two methods tracking the same block might yield different block speed measurements if the duration and distance measurements differ between the two methods.

## Appendix C: Probability of co-occurrence calculation

Co-occurrence is defined as the overlap between blocked regions detected by two or more different methods, which corresponds to a region of $C_k=2$ in similarity calculations. We quantified co-occurrence by counting the time steps in which there was overlap ($n[M1\cap M2]$) during the similarity calculations as well as the number of blocks for each of the methods (*n*[*M*1], *n*[*M*2], etc), then finding $\min (n[M1],n[M2],n[M1\cap M2])$ per time step.

The probability of co-occurrence is defined as

10 $$\begin{aligned} P(M1|M2) = \frac{P(M1\cap M2)}{P(M2)}, \end{aligned}$$

which translates to the number of times that blocks detected by methods M1 and M2 overlap over the number of times that blocks detected by method M2 occur.

## Appendix D: Spatial similarity calculation

This method is similar in concept to the Jaccard index (Jaccard 1908), also known as intersection over union:

11 $$\begin{aligned} J(A,B) = \frac{A\cap B}{A\cup B} = \frac{A\cap B}{A+B-A\cap B}, \end{aligned}$$

where $A\cap B$ are the common points between *A* and *B* and $A\cup B$ are the sum total of points in *A* and *B*, minus the number of common points. In this instance, spatial similarity calculations are performed on two fields *M*1 and *M*2, consisting of regularly spaced grid points whose values are either 1 (block) or 0 (not a block) according to the corresponding objective detection method.

However, there are two points to consider in these calculations:

1. We only wish to count common presence, not common absence (in order to quantify the amount of agreement when two methods detect a block).
2. A simple count of commonly blocked gridpoints between two methods will overemphasize spatial agreement at higher latitudes, since the meridians converge at the poles and the actual distance between gridpoints is shorter.

To address point 1, the two fields are summed together ($F=M1+M2$), where gridpoints have values of 0 (no detection), 1 (one method detects blocking), or 2 (both methods detect blocking); only nonzero gridpoints in *F* are considered.

For each cluster $C_k$ of contiguous nonzero gridpoints on *F*

12 $$\begin{aligned} S(C_k) = \frac{\sum \nolimits _{n=1}^{P_k}\left( v_n-1\right) \cos (\phi )_n}{\sum \nolimits _{n=1}^{P_k}cos(\phi )_n}, \end{aligned}$$

where $P_k$ is the number of points in $C_k$, $v_n$ is the value of the gridpoint (either 1 or 2), and $cos(\phi )_n$ is the cosine of that gridpoint’s latitude value.

Summing the cosine latitude values of gridpoints, rather than the number of points in $C_k$, addresses point 2, as $\cos (\phi )_n$ is smaller at higher latitudes (thus approximating the smaller area between gridpoints). The numerator is essentially the intersection of blocked points ($M1\cap M2$); the sum will only include $\cos (\phi )_n$ where $v_n=2$ (since $v_n=1$ means that $\cos (\phi )_n$ is multiplied by 0). The denominator is the union ($M1\cup M2$), where all $\cos (\phi )_n$ are included in the sum.

## Appendix E: Significance testing

The quantities in Sect. 3 are considered to be statistically significant if $p<0.05$ (and highly significant if $p<0.01$), which implies that the quantity does not satisfy the null hypothesis. The following sections explain the methods used to establish significance.

### E.1: Permutation test

The permutation test is a nonparametric method of hypothesis testing similar to bootstrapping (but without replacement). A sampling distribution is constructed by resampling and/or shuffling data, thus avoiding assumption of a known sampling distribution (for example, a normal distribution).

The general methodology is as follows:

1. Calculate the reference statistic *R* (correlation, difference of medians, etc.) between the two datasets
2. Construct the sample distribution *D* by repeating the following 10,000 times:
  - Generate samples *S*1 and *S*2 (specifics in subsequent subsections).
  - Repeat the calculation of *R* using *S*1 and *S*2 to generate sample statistic $\widetilde{R}.$
3. Count the number of times, $n_D$, in *D* where $\widetilde{R}$ is more extreme than *R*:
  13 $$\begin{aligned} n_D = {\left\{ \begin{array}{ll} \text {number of } \widetilde{R}\ge R,&\quad \text {if } R>D_{50}\\ \text {number of }\widetilde{R}\le R &\quad \text {otherwise}, \end{array}\right. } \end{aligned}$$
  where $D_{50}$ representing the 50th percentile of *D* and $n_D$ representing the number of instances in *D* which satisfy the appropriate case.

The p value is calculated as

14 $$\begin{aligned} p=\frac{n_D}{10{,}000}. \end{aligned}$$

#### E.1.1: Blocking duration, zonal distance traveled, size, speed (Sect. 3.3)

*Null hypothesis* Vectors of values *V*1 and *V*2 are drawn from the same distribution. Therefore, if *V*1 and *V*2 are pooled and two new samples *S*1 and *S*2 are drawn from that common pool, the difference in medians, *M*(*S*1, *S*2), of the samples’ distributions should be similar to *M*(*V*1, *V*2).

*Alternate hypothesis* The distributions of *V*1 and *V*2 are different enough that *M*(*V*1, *V*2) will differ significantly from *M*(*S*1, *S*2)

*Sampling method*

1. Combine all values from *V*1 and *V*2 into common pool *P*.
2. Draw new samples *S*1 (same length as *V*1) and *S*2 (same length as *V*2) from *P* without replacement and calculate *M*(*S*1, *S*2).

#### E.1.2: Pattern correlation (Sect. 3.4)

*Null hypothesis* For fields *F*1 and *F*2, correlation *C*(*F*1, *F*2) will not change significantly if one of the grids is shuffled (implying that the correlation value would be similar if the pattern was entirely random).

*Alternate hypothesis**C*(*F*1, *F*2) is a result of this particular arrangement of frequency values in each of the two grids being compared.

*Sampling method*

1. Generate a list of 1000 random array indices *i*.
2. Create two array subsamples, $S1=F1[i]$ and $S2=F2[i]$
3. Shuffle the order of *S*2 to create $\widehat{S2}$ and calculate $C(S1,\widehat{S2})$.

### E.2: Probability of co-occurrence (Sect. 3.4)

In order to verify that the probabilities would not significantly change with the addition of more data, we took samples of the entire dataset for a particular region and season, with the samples consisting of the equivalent of $n=\{1,2,\ldots ,length(data)\}$ years that were randomly selected from the set of possible years in the ERA-Interim data; probability of co-occurrence was re-calculated with those samples. This procedure was repeated 1000 times and stored as pairs of years and probability (*n*, *P*).

We calculated a linear regression between *n* and each of the probability values ($lm(n\sim P(M1|M2))$ and $lm(n\sim P(M2|M1))$) using the linear model (lm) function in R. Since the slope of the line was approximately zero, the estimated equation was effectively the coefficient, which equaled the initially calculated probability value. The lm function returns a p value for each coefficient, therefore giving us a p value for each calculated probability.

### E.3: Spatial similarity (Sect. 3.4)

This methodology was similar to the permutation test, although the end calculation for the p value was slightly different.

*Null hypothesis* The algorithms detect features equivalently. Therefore, for fields *F*1 and *F*2, *S*(*F*1, *F*2) will not change significantly if the values of gridpoints on *F*1 and *F*2 are pooled and randomly reassigned.

*Alternate hypothesis**S*(*F*1, *F*2) is dependent upon the results of the two algorithms being compared.

For each pair of overlapping blocks, for the set of all nonzero gridpoint coordinates *G* in both *F*1 and *F*2, the following is repeated 100 times:

1. Determine the number of blocked grid points in *F*1 and *F*2, *nF*1 and *nF*2.
2. Set all *F*1[*G*] and *F*2[*G*] to 0.
3. Randomly reassign *nF*1 grid points in *F*1[*G*] a value of 1 to create *S*1; randomly reassign *nF*2 grid points in *F*2[*G*] a value of 1 to create *S*2.
4. Calculate *S*(*S*1, *S*2) and store the new value.

The result is two distributions; the distribution of calculated similarity values, *S*, and the distribution of the sampled similarity values, $\widetilde{S}$. The difference between the two distributions is established using the Wilcoxon test (wilcox.test in R), which returns the p value.
